# Metagenomic tracking of antibiotic resistance genes through a pre‐harvest vegetable production system: an integrated lab‐, microcosm‐ and greenhouse‐scale analysis

**DOI:** 10.1111/1462-2920.16022

**Published:** 2022-05-18

**Authors:** Ishi Keenum, Lauren Wind, Partha Ray, Giselle Guron, Chaoqi Chen, Katharine Knowlton, Monica Ponder, Amy Pruden

**Affiliations:** ^1^ Department of Civil and Environmental Engineering Virginia Tech Blacksburg VA USA; ^2^ Department of Biological Systems Engineering Virginia Tech Blacksburg VA USA; ^3^ Department of Animal Sciences, School of Agriculture, Policy and Development University of Reading Reading RG6 6AR UK; ^4^ Department of Food Science and Technology Virginia Tech Blacksburg VA USA; ^5^ Department of Crop and Soil Environmental Sciences Virginia Tech Blacksburg VA USA; ^6^ Department of Dairy Science Virginia Tech Blacksburg VA USA

## Abstract

Prior research demonstrated the potential for agricultural production systems to contribute to the environmental spread of antibiotic resistance genes (ARGs). However, there is a need for integrated assessment of critical management points for minimizing this potential. Shotgun metagenomic sequencing data were analysed to comprehensively compare total ARG profiles characteristic of amendments (manure or compost) derived from either beef or dairy cattle (with and without dosing antibiotics according to conventional practice), soil (loamy sand or silty clay loam) and vegetable (lettuce or radish) samples collected across studies carried out at laboratory‐, microcosm‐ and greenhouse‐scale. Vegetables carried the greatest diversity of ARGs (*n* = 838) as well as the most ARG‐mobile genetic element co‐occurrences (*n* = 945). Radishes grown in manure‐ or compost‐amended soils harboured a higher relative abundance of total (0.91 and 0.91 ARGs/16S rRNA gene) and clinically relevant ARGs than vegetables from other experimental conditions (average: 0.36 ARGs/16S rRNA gene). Lettuce carried the highest relative abundance of pathogen gene markers among the metagenomes examined. Total ARG relative abundances were highest on vegetables grown in loamy sand receiving antibiotic‐treated beef amendments. The findings emphasize that additional barriers, such as post‐harvest processes, merit further study to minimize potential exposure to consumers.

## Introduction

Animal manures have been identified as rich in antibiotic resistance genes (ARGs) (Binh *et al*., 2008; Ma *et al*., 2016; Graham *et al*., 2019) and concerns have arisen regarding their corresponding application in agricultural production systems (Pruden *et al*., 2013; Xie *et al*., 2018). Widespread soil application of animal manure and associated amendments (e.g. compost) leads to questions about the potential for prior antibiotic use in animal husbandry to spur the dissemination of ARGs along the farm to fork continuum, including into the edible portions of plants (Heuer *et al*., 2011a; Marti *et al*., 2013; Yang *et al*., 2014a; He *et al*., 2016; Williams‐Nguyen *et al*., 2016). Along this continuum, numerous variables could enhance or impede ARG dissemination.

There is growing interest in composting, a common practice for attenuating pathogens (Mitchell *et al*., 2015), as a means to also potentially reduce ARGs prior to land application. Prior studies indicate that relative abundance of total ARGs, measured by metagenomics or high‐throughput quantitative polymerase chain reaction (qPCR), tend to decrease during composting (Sharma *et al*., 2009; Zhang *et al*., 2020; Keenum *et al*., 2021). However, the species of animal generating the manure influences the degree of decrease achieved (Qian *et al*., 2018). Furthermore, the abundance of ARGs can become elevated in soil when amended with manure from antibiotic‐dosed animals (Heuer *et al*., 2011b; He *et al*., 2014; Wepking *et al*., 2017). Additionally, long‐term application of manure has been shown to increase plasmid uptake in soil bacterial communities by up to 100%, indicating that ARGs may be transferred at an increased rate (Musovic *et al*., 2014). Soil type can also influence the resistome (i.e. total ARG) trajectory following addition of manure‐derived amendments (Chen *et al*., 2019). Wang *et al*. (2020b) compared three soil types and found that low silt content and pH were strongly associated with higher ARG abundances in soil after long‐term dairy cattle and chicken manure application. In crop production systems, vegetable type (e.g. root vs. leafy) and soil type can also shape the microbiome (Marschner *et al*., 2001) and resistome (Sun *et al*., 2019). In a field study, Fogler *et al*. (2019) found that lettuce and radishes grown in soil amended with manure‐derived fertilizers carried greater abundances of total ARGs than when grown in soil amended with inorganic chemical fertilizers.

Given the multitude of factors that can shape the resistomes in crop production systems, it is important to develop an understanding of how these factors interact to shape the ultimate resistome carried by resulting food products. This is especially the case for vegetables eaten raw, such as lettuce and radishes, because of the greater potential for humans to be exposed to antibiotic‐resistant bacteria and ARGs via consumption (Hölzel *et al*., 2018). Understanding mechanisms shaping resistomes is also important. For example, it is often observed that shifts in bacterial community composition are a primary driver of corresponding shifts in abundances and types of ARGs observed, as has been reported in soils (Yang *et al*., 2014b). On the other hand, when resistome compositions shift across samples in a manner that is distinct from the patterns of bacterial taxonomic community shifts, horizontal gene transfer of ARGs across taxa could be a key driver. Thus, the mobility of the resistome is also a concern in assessing the potential risk for antibiotic resistance spread in agricultural production systems (Martínez *et al*., 2015). Tracking mobile genetic elements (MGEs), mobility of ARGs, and their potential carriage in human pathogens can help to identify critical management points for the spread of antibiotic resistance in crop production systems and to prioritize mitigation efforts.

Different conclusions can be drawn when assessing the potential for various agricultural practices to stimulate or attenuate antibiotic resistance, depending on which ARG is targeted by qPCR (Storteboom *et al*., 2007; Wang *et al*., 2015). Shotgun metagenomic sequencing circumvents this problem by randomly sequencing DNA fragments across the microbial community, which can then be compared to online databases to identify ARGs, MGEs and taxonomic markers (Tringe and Rubin, 2005; Cytryn, 2013). Using direct annotation, the prevalence of ARGs across environments can be assessed. With assembly of the data, prevalence of co‐occurrent ARGs and MGEs can be tracked to identify potentially efficient and effective opportunities for minimizing the spread of antibiotic resistance in crop production systems.

Recently we evaluated individual hypothetical critical management points for the spread of antibiotic resistance in preharvest vegetable production systems; including antibiotic use in beef and dairy cattle (Ray *et al*., 2017), cattle manure composting (Keenum *et al*., 2021), amendment of manure versus compost to soil (Chen *et al*., 2019), wait period following amendment application (Chen *et al*., 2018b) and root (i.e. radish) versus leafy (i.e. lettuce) vegetable cultivation (Guron *et al*., 2019). In general, it was found that composting significantly reduced ARGs compared to untreated manure. Furthermore, the relative abundance of total ARGs was lower in compost‐amended than manure‐amended soils after 120‐day incubation in microcosms (Chen *et al*., 2019). However, there was no difference in impact of compost versus manure amendment on the relative abundance of total ARGs carried by the vegetables. Interestingly, there were significant differences in the relative abundance of total ARGs carried by lettuce versus radish (Guron *et al*., 2019). The relative abundance of total ARGs on radishes grown in soil amended with manure or compost was greater than on lettuce or on radish amended with chemical fertilizers. The effects of administered antibiotics (intramammary dosed pirlimycin in dairy cows and in feed chlortetracycline, sulfamethazine and tylosin in beef steers) were difficult to discern in many conditions. Integrated evaluation across the hypothetical control points captured by these studies is needed in order to rank their relative importance in contributing to the spread of ARGs and where mitigation efforts are likely to be most effective.

**Fig. 1:**
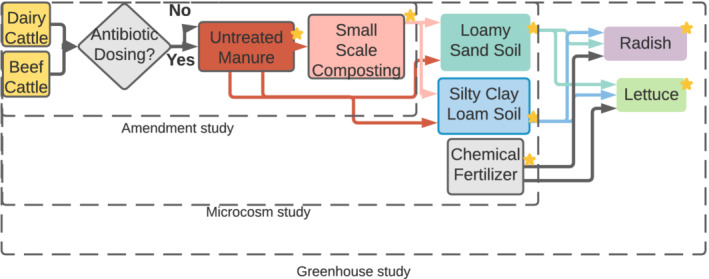
Experimental design and sampling schematic for all conditions. Stars indicate sampling points. Replicate numbers and SRA numbers can be found in Tables S1 and S2 respectively.

The objective of this study was to perform an integrated analysis of the resistomes across prior controlled lab‐, microcosm‐ and greenhouse‐scale studies, which employed the same manure‐derived amendments and soils throughout. Specifically, metagenomes of 200 samples (including 84 new metagenomes not examined in previous studies) representing 23 experimental conditions and time points were examined in replicate across three sample types: amendment, soil and vegetable (Fig. 1). This combined analysis of new data alongside data published in prior studies focused on individual critical management points (i.e. effects of antibiotic administration, composting, soil type and vegetable type) enabled a broader and more holistic assessment of their relative effects on vegetable resistomes and sources of ARGs along the farm to fork continuum. ARG mobility and clinical relevance were also tracked. The amendments consisted of raw or composted manure derived from beef or dairy cattle, with half collected during antibiotic administration (with antibiotics) and the other half collected from cattle not administered antibiotics (control) (Keenum *et al*., 2021). The resulting manure or compost was applied to two soil types (loamy sand and silty clay loam) in microcosms and incubated for 120 days (Chen *et al*., 2019), to simulate a harvest wait period, or subject to cultivation of radishes or lettuce in a greenhouse (Guron *et al*., 2019). A no amendment control was applied in the microcosms and a chemical fertilizer only control was applied for the greenhouse study. Short reads assembly and annotation were applied with stringent criteria to identify the similarities and differences in mobile ARGs (i.e. ARGs that co‐occur with MGEs on same contigs). This systems‐level assessment can help to identify and prioritize where mitigation efforts are likely to be most effective for limiting the spread of antibiotic resistance from farm‐to‐fork.

## Experimental procedures

### Manure, compost, soil and vegetable samples

This study examined shotgun metagenomic datasets to profile and track ARGs across two parallel lettuce and radish pre‐harvest production systems (Fig. 1). This was achieved through integrated analysis of previously published manure and compost (Ray *et al*., 2017; Keenum *et al*., 2021) amended soil microcosm (Chen *et al*., 2019), and greenhouse vegetable cultivation (Guron *et al*., 2019) studies, with an additional 84 previously unpublished metagenomes added in order to include beef manure‐derived amendment conditions in the soil microcosm and radish greenhouse dimensions of the study (Table S1).

Manure was procured from 18 individually housed beef steers and dairy cows selected for their respectively similar body weights, history of antibiotic use (none for steer, none in previous lactation cycle for cows) and consistent stage of lactation (Ray *et al*., 2017). Three steers were fed 350 mg of chlorotetracycline and sulfamethazine per day, three were fed 11 mg tylosin per kg feed, and three were fed a non‐medicated diet. Three peak lactation cows received no antibiotics, three peak lactation cows were treated with two intermammary doses of 50 mg pirlimycin; and three cows at the end of lactation received a single intermammary dose of 300 mg cephapirin per quarter (i.e. 300 × 4 = 1200 mg per cow), according to standard veterinary practice. Faeces and urine were composited to obtain a homogenous mixture of ‘with antibiotic’ and ‘control’ manure for both beef and dairy cattle (i.e. four distinct manures for subsequent composting) and to simulate the possibility of segregating antibiotic‐containing manures as a management practice.

The amendment treatments included: (i) control compost, (ii) compost with antibiotics, (iii) control manure and (iv) manure with antibiotics. Composting of the generated manure was carried out at small‐scale, 0.23 m^3^, to enable parallel comparison of multiple conditions, at Kentland Farm in Blacksburg, VA, USA (Ray *et al*., 2017; Keenum *et al*., 2021). In brief, 20–22 kg of manure was composted over 42 days using sawdust, hay and grass to maintain a thermophilic temperature of 55°C for 3 days. Manure and composts were sampled in triplicate at time 0 d. The compost was additionally sampled at time 4 d (thermophilic temperature reached) and 42 d (end of compost curing), which resulted in 31 metagenomic samples for the current analysis.

For the soil study, detailed microcosm design and soil types were described previously (Chen *et al*., 2018a). In brief, a loamy sand and a silty clay loam topsoil (0–5 cm) were collected in 2016 from three farms in Virginia. The soil microcosms consisted of triplicate glass jars containing soils amended with the small‐scale generated manure or composts described above at a typical agronomic nitrogen application rate (Evanylo, 2009). The soil treatments included: (i) dairy control compost, (ii) dairy compost with antibiotics, (iii) dairy control manures, (iv) dairy manure with antibiotics, (v) beef control compost, (vi) beef compost with antibiotics, (vii) beef control manures, (viii) beef manure with antibiotics and (ix) an inorganic fertilizer control. Microcosms were incubated under aerobic conditions, in the dark, at 20°C, and with soil moisture maintained at 50% field capacity by watering one time weekly with sterile water. Microcosms were sacrificed in triplicate for each amended soil collection on day 1 and 120 after set up, which resulted in 78 metagenomic samples for the current analysis.

Radish and lettuce were grown in a parallel greenhouse study, using the same amendments generated from the compost study (Ray *et al*., 2017; Keenum *et al*., 2021) and the same soils from the soil microcosm study (Chen *et al*., 2019). The detailed methods of vegetable cultivation were previously described by Guron *et al*. (2019). In brief, each round pot (6‐in. diameter, 5‐in. height; Poppelmann GmbH, Lohne, Germany), 0.9 kg (dry weight) of each soil type (silty clay loam and loamy sand) was mixed by hand with the amendments (*n* = 20): (i) dairy control compost, (ii) dairy compost with antibiotics, (iii) dairy control manures, (iv) dairy manure with antibiotics, (v) beef control compost, (vi) beef compost with antibiotics, (vii) beef control manures, (viii) beef manure with antibiotics and inorganic fertilizer controls (*n* = 10). Triplicate pots of each condition were mixed and cultivated in a greenhouse at Virginia Tech (Blacksburg, VA) and appropriate radish seed and lettuce transplants were sown into the soil to analyse the effects of root versus leafy vegetable surface resistomes (Guron *et al*., 2019). Municipal drinking water (Blacksburg, VA) was filtered using granulated activated carbon to remove disinfectants and pots were watered by hand to maintain 50%–70% field capacity moisture. Water‐soluble 20‐20‐20 fertilizer (JR Peters, Allentown, PA, USA) was applied to all pots as necessary to maintain plant health. Hairnets were used to cover pots during growth as a barrier to cross‐contamination. Lettuce leaves and radishes were harvested after 34 and 60 days respectively, which resulted in 91 metagenomic samples for the current analysis. Amendment, soil and vegetable samples were stored at −80°C until downstream molecular analysis.

### DNA extraction and metagenomic sequencing

DNA was extracted from 0.5 g of soil, manure, or compost. Taproots or leaves were weighed in 710‐ml sterile filter bags with peptone buffer (Becton, Dickinson and Company, Franklin Lakes, NJ, USA) containing Tween 80 (Fisher Scientific, Waltham, MA, USA) (0.1% each) at a 1/10 (wt./wt.) dilution (Guron *et al*., 2019). Bacteria were removed from the vegetable surfaces by shaking at 220 rpm for 5 min using a benchtop rotator, alternating with hand‐massaging for 2 min and shaking again for an additional 5 min. The suspensions were filtered through a 0.22‐μm 47‐mm mixed cellulose ester membrane (EMD Millipore, Burlington, MA, USA) to collect microbial cells dissociated from the vegetable surfaces. In addition to samples, DNAase free water was filtered through 0.22‐μm filters to capture any laboratory‐specific contamination and these samples were considered filter blanks. The filters were folded, torn, transferred to Lysing Matrix E tubes (FastDNA Spin kit for Soil, MP Biomedicals, Solon, OH, USA) and stored at −80°C until extraction. All DNA was extracted and eluted in 100 μl water and subjected to OneStep PCR Inhibitor Removal (Zymo Research Corporation, Irvine, CA, USA) before storing at −80°C. Extraction followed the manufacturer's instructions, except that a 2‐h incubation period was added to the protocols immediately following the bead‐beating step to optimize lysis of microbial cells and the final centrifugation step was extended to 3 min to maximize capture of DNA.

Shotgun metagenomic sequencing, including TruSeq library construction, was carried out across three lanes at the Biocomplexity Institute of Virginia Tech (BI, Blacksburg, VA, USA), using an Illumina HiSeq 2500 in high output mode with a paired‐end 2 × 100 read length protocol. Fastq files were obtained from the listed NCBI BioProjects: PRJNA506850 (Keenum *et al*., 2021), PRJNA489261 (Chen *et al*., 2019), SRP151152 (Guron *et al*., 2019) [Sequence Read Archives (SRA) listed in Table S2]. An additional lane of sequencing was performed at the Duke Centre for Genomics Sequencing Centre (Duke; Durham, NC, USA) to include additional conditions and enhance replication for the purpose of the present study. These samples and conditions are denoted in Fig. 1. Filter blank DNA extracts yielded insufficient DNA for metagenomic sequencing. These were sequenced on a full flow cell using an Illumina NovaSeq 6000 with paired‐end 2 × 150 read length protocol. Merged DNA sequences were deposited in the National Centre for Biotechnology Information NCBI SRA (Table S3). Vegetable samples had chloroplasts removed in the same manner as in Guron *et al*. (2019), but were not rarefied prior to assembly to avoid loss of data.

Metagenomic reads were trimmed with Trimmomatic (Bolger *et al*., 2014) and merged using vsearch (Rognes *et al*., 2016). ARGs were annotated using DIAMOND (Buchfink *et al*., 2015) (amino acid identity ≥80%; *e*‐value cutoff = 1*e*‐10; minimum alignment length = 37 amino acids) against the Comprehensive Antibiotic Resistance Database (Jia *et al*., 2016) (CARD, version 3.0.8) and point mutation genes were removed (Table S4). To account for variable read and gene lengths, gene counts were normalized to 16S rRNA gene copy number with bowtie2 (Langmead and Salzberg, 2012) to report relative abundance using Greengenes (DeSantis *et al*., 2006; Li *et al*., 2015a). Metagenomic coverage was estimated for each sample using Nonpareil 3 (Rodriguez‐R *et al*., 2018). Taxonomy was assessed using kraken2 (Wood *et al*., 2019) with the plasmid removed NCBI's Reference Sequence Database (O'Leary *et al*., 2016; Doster *et al*., 2019) and pathogens were assessed by membership to the species listed in the Bad Bug Book (US Food and Drug Administration, 2004). Clinically relevant ARGs were defined as those most relevant to difficult to treat resistant infections found in humans (Majeed *et al*., 2021), as summarized in Table S5.

Reads were assembled using MEGAHIT (Li *et al*., 2015b) and annotated for ARGS using a compilation of multiple ARG databases (ARDB, CARD, MEGARes, SARG and DeepARG‐DB (Liu and Pop, 2009; Jia *et al*., 2016; Lakin *et al*., 2017; Arango‐Argoty *et al*., 2018; Yin *et al*., 2018). MGEs were identified from the NCBI non‐redundant database by using a keyword search (Forsberg *et al*., 2014). Thus, genes related to any of the following keywords – transposase, transposon, integrase, integron and recombinase – were classified as MGEs. Scaffolds were annotated using DIAMOND (Buchfink *et al*., 2015) with an identity cutoff of 60% and an *e*‐value cutoff of 1 × 10^−10^. A best‐hit approach was applied for each location. Co‐occurrences were determined by using the annotation files produced by MetaCompare (Oh *et al*., 2018). ARG and MGE co‐occurrences were filtered to ensure annotations were not due to database overlap (same gene occurring in both CARD and keyword search resulting in misrepresentation of gene). A co‐occurrence was considered as relevant if it was found to occur in the majority of the replicates for a condition (e.g. two out of three Radish compost samples fertilized with dairy manure with antibiotics).

### Statistical analysis

ANOSIM and non‐metric multidimensional scaling plots were performed to compare resemblance data of ARG classes (Bray–Curtis). Due to the lack of normal distribution, one‐way comparisons of the relative abundances for each ARG class were performed using R functions for Wilcoxon rank‐sum test (between two levels of soil textures), Kruskal–Wallis test (among three levels of amendment types) and Dunn's test for *post hoc* examination. The Bonferroni method was applied for *p‐*value adjustment. Statistical significance was defined at *p* < 0.05.

## Results

### Overview of experimental conditions, samples and data included in this study

Two hundred (116 previously published and 84 new) metagenomes were examined across three sample categories representing key points in the pre‐harvest vegetable production system (31 amendments, 78 soils, 91 vegetables, Fig. 1; Tables S1, S2, S3). Unlike previous examination of these experimental conditions at greenhouse‐scale (Chen *et al*., 2019; Guron *et al*., 2019), this study also examined the effects of cattle type (beef vs. dairy) and corresponding differences in antibiotics typically administered, together with soil type, on the observed vegetable resistomes.

### Overarching influence of soil texture on vegetable resistomes

**Fig. 2:**
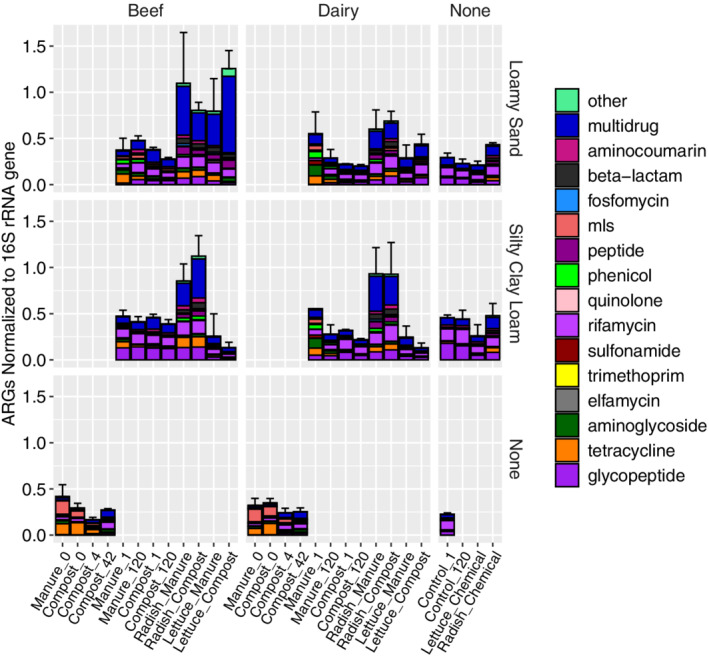
Average relative abundance (normalized to the 16S rRNA gene) of total ARGs annotated to CARD v3.0.7 (minimum identity = 80%, 25 amino acid length, *e*‐value cutoff 1 × 10^−10^) with intrinsic and point mutation genes removed (Table S5). Antibiotic conditions were combined for this figure. Error bars indicate the standard deviation. ‘None’ indicators control chemical fertilizer conditions. Experimental conditions and day sampled are indicated in the sample names on the *x*‐axis and the number of replicates for each condition is described in Table S1. The relative abundance of total ARGs on radishes amended with manure or compost fertilizers was significantly greater than on lettuce (*p* < 0.001) or radish amended with chemical fertilizers (*p* < 0.001). No other total ARG comparisons were statistically significant.

Significant differences were observed in the relative abundance (16S rRNA gene normalized) of total ARGs when comparing across all sample categories [Fig. 2; Fig. S1, amendment (*n* = 31), soil (*n* = 78), vegetable (*n* = 91); Kruskal–Wallis (*p* < 0.001)], as well as in the profiles of the types of ARGs detected [Fig. S2, analysis of similarities (ANOSIM), *R* = 0.65, *p* < 0.001]. Examining the soils specifically, the relative abundance of total ARGs was higher in control (without organic or chemical amendments) silty clay loam soils than loamy sand soils at time zero and after 120 days (Dunn's test, day 1: *p* = 0.02, day 120: *p* = 0.036). No difference in relative abundance of total ARGs was observed when assessing the effect of organic amendments on each of the individual soil textures. However, relative abundance of total ARGs was higher on lettuce grown in loamy sand amended with compost than on lettuce grown in silty clay loam amended with compost (Dunn's test, *p* < 0.001). No significant differences were found in total ARG relative abundance on vegetable surfaces when assessing the effect of organic amendments with and without antibiotics. Also, no notable differences were found in individual antibiotic resistance class abundances based on soil texture or antibiotic usage.

### Vegetables carried higher relative abundances of ARGs than corresponding soils

This study was uniquely able to compare relative abundances of ARGs on vegetables at harvest versus the same soils that had remained fallow in the microcosms without cultivation. The relative abundances of total ARGs carried either by lettuce or radish grown in soil amended with any of the four composts and by radishes fertilized with dairy manure without antibiotics were significantly higher than in soils where no vegetables were grown (Dunn's test, *p* < 0.05 for all cases).

### Effects of beef versus dairy manure and corresponding antibiotic administration regimes on downstream soil and vegetable resistomes

Beef cattle were dosed with chlorotetracycline (tetracycline drug class) and sulfamethazine (sulfonamide class) or tylosin [macrolide–lincosamide–streptogramin (MLS) class]. Dairy cattle were treated with either intermammary doses of pirlimycin (MLS class) or cephapirin (beta‐lactam class). Antibiotic‐treated manures from each cattle type were composited for this study.

ARG profiles were distinct in soil microcosms amended with beef manure or compost relative to corresponding dairy manure or compost‐amended soils (*R* = 0.23, *p =* 0.001, ANOSIM). Additionally, time after amendment and soil type were significant factors in altering the resistome profile in soils amended with beef manure or compost (time: *R* = 0.18, *p =* 0.001, soil type: *R* = 0.49, *p =* 0.001, ANOSIM).

Soil microcosms amended with beef or dairy manure with antibiotics initially contained significantly higher relative abundances of total ARGs than control soils (Dunn's test, *p* = 0.02, *p* = 0.04). This was not the case when amended with control manures (*p* = 0.4, *p* = 0.4). There was no difference in the relative abundance of total ARGs in the manure or compost‐amended soils relative to the inorganic amended controls by day 120. However, soils amended with beef‐derived manures with and without antibiotics contained significantly higher levels of ARGs belonging to the MLS class at day 120 relative to the inorganic‐amended control (Dunn's test, beef manure with antibiotics: *p* = 0.01, beef manure without antibiotics: *p* = 0.01, beef compost with antibiotics: *p* = 0.01, beef compost without antibiotics: *p* = 0.01). No differences were found in soils receiving beef‐derived amendments from day 0 to day 120 for any other antibiotic resistance class.

The relative abundance of total ARGs detected on vegetables grown in beef manure‐amended or beef compost‐amended loamy sand was significantly higher than when grown in dairy manure‐amended or dairy compost‐amended conditions (Wilcoxon, *p* < 0.001). Additionally, the relative abundance of total ARGs on lettuce and radish grown in beef manure with antibiotics‐amended loamy sand were higher than when grown in the organic control (no antibiotic) amended soils (Dunn's test, lettuce: *p* = 0.01, radish: *p* = 0.03). ARGs were not elevated on vegetables when dairy manure‐derived amendments were applied to loamy sand.

### Influence of soil and amendments on vegetable resistome composition

Differences in ARG profile were further observed when examining vegetables (lettuce and radish analysed together) grown in loamy sand based on prior antibiotic administration to cattle and, more specifically, on radishes grown in loamy sand with beef‐derived compost or manure amendments (All Vegetables: ANOSIM, *R* = 0.10, *p* = 0.03, Radishes: *R* = 0.22, *p* = 0.01). No differences were observed in the ARG profiles of vegetables grown in loamy sand receiving beef‐derived amendments based on manure treatment (raw manure vs. composted). Additionally, while the resistome profiles of radish and lettuce were each distinct from soils, radish profiles were more strongly separated from soils than were lettuce profiles (ANOSIM, radish vs. soil: *R* = 0.58, *p* = 0.001, lettuce vs. soil: *R* = 0.30, *p* = 0.001).

### Shared and differential ARGs across experimental conditions

**Fig. 3:**
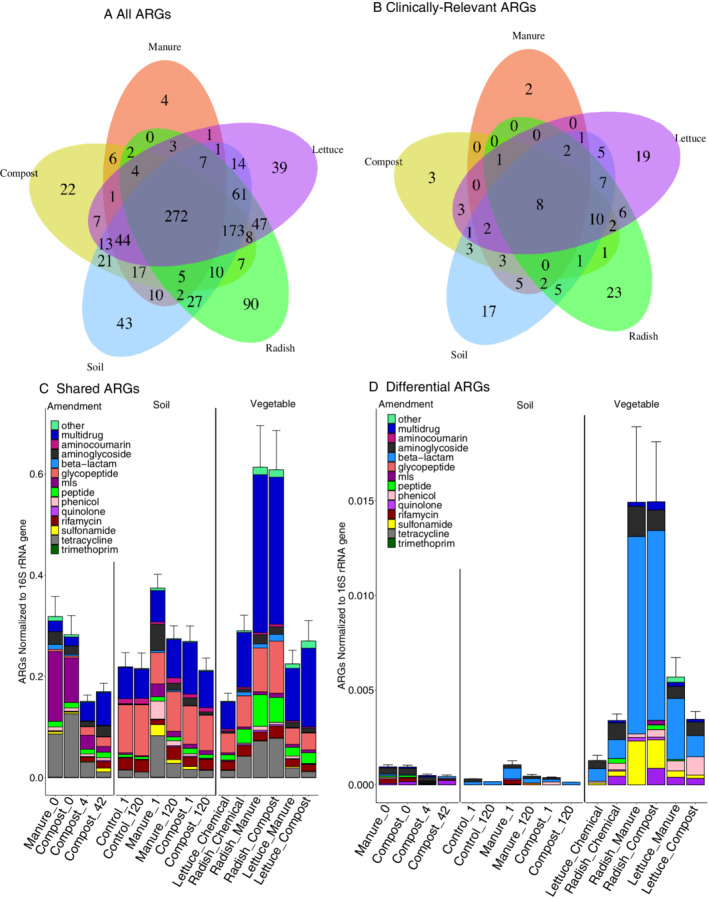
Shared and differential ARGs across sample categories and experimental conditions. Error bars represent the standard deviation in the 16S rRNA gene normalized abundance of ARGs. A. All detected ARGs, (B) clinically relevant ARGs as defined in Table S3. C. Shared ARGs (*n* = 567) coloured by drug class in all sampled environments. D. ARGs unique to each environment (*n* = 256) coloured by drug class in all sampled environments.

Venn diagrams were constructed to identify shared and differential ARGs across sample categories and experimental conditions (Fig. 3A). Fifty‐five percent of ARGs (*n* = 525) were shared across all three sample categories (amendment, soil and vegetable) and 28 % (*n* = 272) were shared across all five experimental conditions (soil, compost, manure, lettuce and radish). Examining further, the majority of the overlapping 525 genes (aside from the 272 found in all samples) were specifically shared between radish, lettuce, soil and compost samples (*n* = 173). Manure amendments shared few ARGs (*n* = 7) with radish, lettuce and soil samples (Fig. 3A). Examining the effects of cattle type and antibiotic dosing on ARG detection across the three sample categories, 50 and 31 unique ARGs were found specifically in the dairy and beef conditions dosed with antibiotic respectively. All of these ARGs were only detected in amendments (raw manure and days 0, 4 and 42 of composting).

Notably, relative abundance of shared ARGs [ARGs found in each of the three categories (*n* = 525)] increased significantly from amendment to soil to vegetable, with the sharpest increase noted when comparing amendments to vegetables (Fig. 3C, Kruskal–Wallis, *p =* 0.03, Dunn's test: *p* = 0.01). No significant differences were observed among ARGs shared across sample types based on prior antibiotic administration. However, significant differences were observed in soil and vegetable resistomes depending on whether amended with beef versus dairy amendment (Dunn's test; soil: *p* = 0.02, vegetable: *p* < 0.001). The profiles of shared ARGs also shifted from amendment to soil to vegetable (ANOSIM, *p* = 0.001, *R* = 0.39). In particular, MLS ARGs were elevated in the amendments compared to vegetables and soils (2× higher; Dunn's test, *p* = 0.001), whereas glycopeptide ARGs were elevated in soils and vegetables relative to amendments (10× higher, Dunn's test, *p* = 0.002, *p* < 0.001). Aminocoumarin (4× higher than amendments, *p* = 0.004), beta‐lactam (4× higher than amendments and soils, *p* = 0.04, *p* < 0.001), fosfomycin (10× higher than soils, *p* < 0.001) and peptide (3× higher than soils, *p* < 0.001) were also all higher on vegetables. In terms of comparing sample sub‐types (Fig. S3), lettuce grown with beef amendments (manure or compost, with and without antibiotics) carried higher levels of shared ARGs than lettuce grown with dairy amendments (manure or compost, with and without antibiotics) grown in the same loamy sand (Dunn's test, compost: *p =* 0.008, manure: *p =* 0.04).

In terms of differential ARGs, i.e. ARGs found in only one sample type (amendment, soil, or vegetable), those carried on vegetables were higher in relative abundance compared to the other two sample categories (Fig. S4, Kruskal–Wallis, *p =* 0.003, Dunn's test: soil to vegetables *p* < 0.001, amendment to vegetables *p* < 0.001). Differential ARGs identified on vegetables primarily belonged to the aminoglycoside, elfamycin, trimethoprim, quinolone and beta‐lactam resistance classes.

### Clinically relevant ARGs

Clinically relevant ARGs were defined as conveying resistance to World Health Organization classified ‘Reserve’ antibiotics [Table S5, (World Health, 2019; Majeed *et al*., 2021)]. One hundred and thirty‐two clinically relevant ARGs were detected across the three sample categories (amendment, soil and vegetable), with the greatest number of clinically relevant ARGs found only in vegetable samples (radish and lettuce) (Fig. 3B; 36%). Of the 48 clinically relevant ARGs detected only on vegetables, 46 belonged to the beta‐lactam class (including *blaKPC‐15* and *blaKPC‐10*; Table S6) and the rest belonged to the peptide resistance class. The beta‐lactamase ARGs varied in sequence composition, aligning to variants including *blaCARB*, *blaCTX‐M*, *blaOXA*, *blaTEM*, *blaSHV* and *blaVIM*. No difference was observed in the number of clinically relevant ARGs detected across amendments, soils and vegetables based on prior antibiotic addition. Radish and lettuce were found to share more clinically relevant ARGs with soil and compost combined (10 ARGs) than with manure‐amended soils (one ARG). Radishes carrying clinically relevant ARGs were found in multiple conditions (i.e. soils amended with manure or compost, with or without antibiotics).

Remarkably, a greater number of clinically relevant ARG types were uniquely found only in radish (23) or lettuce (19) samples, where manure and compost carried surprisingly few unique clinically relevant annotations (2 and 3 respectively). Furthermore, radish and lettuce had strikingly more clinically relevant ARG annotations in common with soil samples (35, 36 respectively) than with the manure and compost amendments combined (26, 18 respectively).

Eight clinically relevant ARGs were found in all sample categories (*blaCARB‐5*, *blaMCR‐4*.*2*, *blaMCR‐8*.*1*, *blaOXA‐226*, *blaOXA‐278*, *blaOXA‐296*, *blaOXA‐347*, *vanA*) and more (*n* = 10) clinically relevant ARGs were shared across vegetables, compost and soil than vegetables, manure and soil (*n* = 2). This difference in shared clinically relevant ARGs suggests that compost may transfer more ARGs to vegetable surfaces than manure amendments.

### Profiling of putative pathogen gene markers

**Fig. 4:**
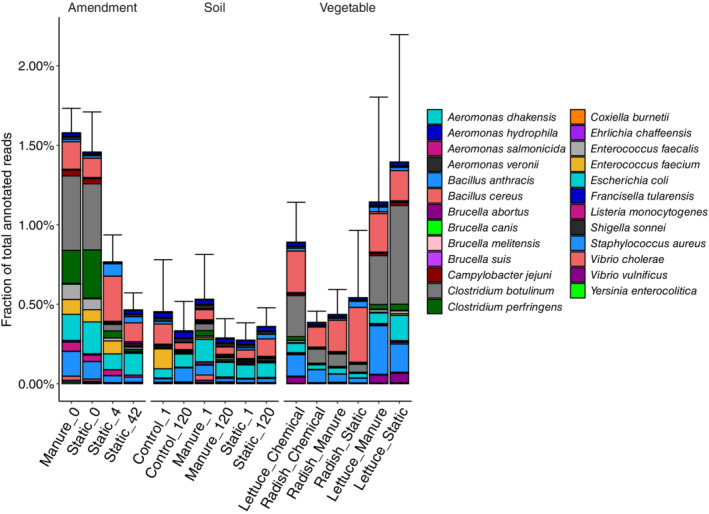
Average detected reads in manure, soil and vegetables annotated to species known to contain strains of produce‐relevant pathogens, as defined in the Bad Bug Book (US Food and Drug Administration, 2004). Reads were aligned to the NCBI RefSeq database with a hash table and least common ancestor approach (Wood *et al*., 2019) and only those annotated to the species level were included. Plasmid annotated markers were removed (Doster *et al*., 2019). Stacked bars represent averages across replicates and error bars represent the standard deviation in the fraction of annotated reads. Lettuce samples had significantly higher total fractions of reads annotated to bacterial species containing pathogenic strains in all conditions than radishes. Composting was significantly associated with decreased pathogen gene markers.

Metagenomes were further searched to identify taxonomic markers for potential pathogens. Focusing on produce‐relevant species as defined in the Bad Bug Book (US Food and Drug Administration, 2004), corresponding taxonomic markers, as included in the Kraken least common ancestor database (Wood and Salzberg, 2014), were annotated across amendment, soil and vegetable samples by aligning to the non‐plasmid‐associated sequences in the National Centre for Biotechnology Information (NCBI) RefSeq database and employing a least common ancestor approach (Doster *et al*., 2019; Wood *et al*., 2019) (Fig. 4). Notably, reads annotated as belonging to *Clostridia botulinum*, a pathogen that can cause botulism in rare cases, were detected in amendment manure and static compost day 0 as well as in all radish conditions and at least one sample in all lettuce conditions. Gene markers corresponding to *Escherichia coli* were readily found in soils as well as on lettuce, both in conditions with and without manure‐derived amendments. Composting reduced the abundance of gene markers pertaining to taxonomic groups containing pathogens from day 0 to day 42 (Wilcoxon, *p* < 0.001). In contrast to ARG detection on vegetables, more pathogen gene markers were observed on lettuce than on radishes (Wilcoxon, *p* < 0.001). Notably, there was no difference in the carriage of taxonomic markers annotated as pathogens for lettuce grown in soils receiving inorganic versus manure‐derived amendments (Dunn's test, *p >* 0.05 for all combinations).

Nine taxa containing pathogens (*Bacillus cereus*, *Clostridium botulinum*, *Escherichia coli*, *Enterococcus faecalis*, *Yersinia enterocolitica*, *Aeromonas veronii*, *Aeromonas hydrophila*, *Aeromonas salmonicida*, *Staphylococcus aureus*) were found to be in common across all sample categories but only comprised 8%–17% of the total reads mapped to pathogen‐containing taxa (Ranging between 1 and 185 131 reads per sample per pathogen). All taxa found on lettuce that contain pathogenic members were also found in the other sample categories. Six taxa containing pathogens (*Clostridium perfringens*, *Vibrio vulnificus*, *Francisella tularensis*, *Campylobacter jejuni*, *Listeria monocytogenes* and *Enterococcus faecium*) were found only in lettuce, soil, compost and manure samples, but not on radishes. This suggests that lettuce possesses greater potential to carry pathogens from manure‐derived amendments to vegetables. Two taxa containing pathogens (*Brucella melitensis* and *Brucella abortus*) were found in compost, soil, lettuce and radishes and one taxon containing pathogens (*Yersinia enterocolitica*) was exclusive to manure, soil, lettuce and radishes. This indicates that there was little difference in compost or manure amendments contributing potential pathogens to downstream vegetables.

### Co‐occurrence of ARGs and MGEs on assembled contigs

Metagenomic data were assembled to identify ARGs that likely co‐occur with MGEs and thus are more likely to be able to mobilize to new bacterial hosts. Examining annotated contigs for ARGs and MGEs, 3533 unique combinations of ARGs and MGEs were found in at least two of three replicates across all experimental conditions. Of these unique co‐occurrences, 561 were found in more than one sample category (amendment, soil, vegetable), representing 112 unique ARGs belonging to 14 differing antibiotic resistance classes.

**Fig. 5:**
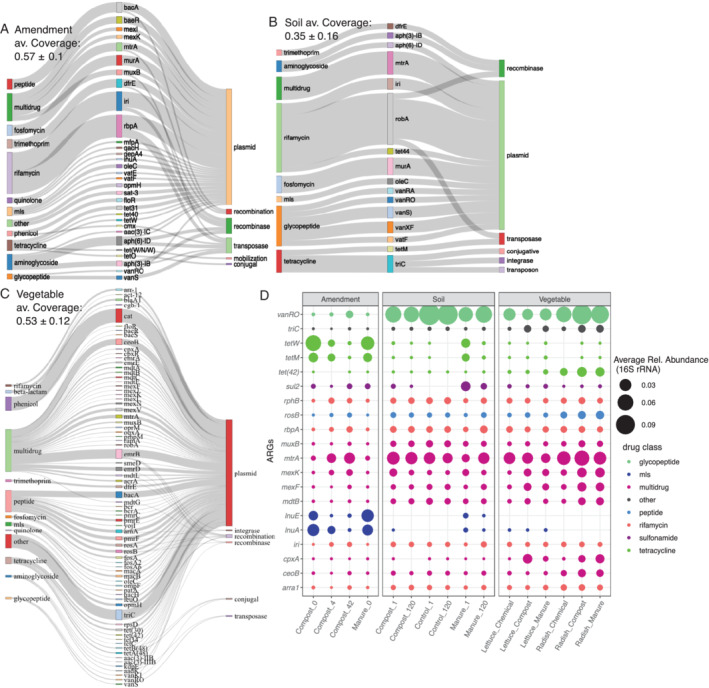
ARGs and MGEs identified on the same assembled contig for each sample category (A) amendment, (B) soil, (C) vegetable. Only contigs that were found in at least two of three samples for each experimental condition, as annotated by MetaCompare using CARD for ARGs and MGEs using an integrated MGE databases [NCBI search for ‘integron’ and I‐VIP (Zhang *et al*., 2018)], were included in the analysis, (D) Top 20 ARGs detected on contigs containing MGEs, as annotated directly to CARD v 3.0.7 (minimum identity = 80%, 25 amino acid length, *e*‐value cutoff 1 × 10^−10^) with point mutation genes removed (Table S5) as an estimate of relative abundance (16S rRNA gene normalized) of mobile ARGs.

Each sample category differed in terms of the number of ARG‐MGE co‐occurrences found, with 80 in amendments, 90 in soils and 391 in vegetables (Fig. 5A–C). The number of co‐occurrent contigs was greater in vegetable samples than in either amendment or soil samples (Kruskal–Wallis; *p =* 0.007). In terms of sample sub‐types (Table S6), day 42 compost (beef and dairy combined) without antibiotics had more co‐occurrences compared to day 42 compost (beef and dairy combined) with antibiotics (Wilcox rank‐sum, *p* < 0.001). However, across both composts, day 42 contained the most ARG‐MGE co‐occurrences, and increases were observed with respect to ARG‐MGE co‐occurrences from day 0 to day 42 (Kruskal–Wallis, *p* < 0.001).

Examining vegetable sub‐types, radishes grown in soils amended with manure or compost carried more co‐occurrences of ARGs than radishes grown with inorganic fertilizers or lettuce (any condition). Radishes grown in loamy sand soil amended with manure containing antibiotics carried the most co‐occurrences among radishes grown under any conditions.

No ARGs co‐occurred with the same MGEs across all three sample categories, but many broader antibiotic resistance classes were consistently found to be associated with MGEs. Co‐occurrences of *dfrE*, *mtrA*, *vanSA*, *murA* and *vanXF* with plasmid marker genes were the most frequently encountered in amendments. These same ARGs were also found in soil, but additional ARGs (*iri*, *rbpA*, *bacA* and *baeR*) occurred at the same rate with plasmid marker genes. In vegetable samples, the most frequently co‐occurring with plasmid marker genes were *dfrE* and *mtrA* and *bacA*. Among ARGs that co‐occurred with multiple types of MGEs in all environments, *aph(6)‐ID*, *ermB* co‐occurred with plasmid marker genes, recombinases and transposases and *tet(M)* co‐occurred with integrases, transposons, plasmids, and conjugative MGEs. Additionally, *dfrE* co‐occurred with recombinase and plasmid marker genes, while *ermB* co‐occurred in vegetables with integrases, recombinases and plasmid marker genes. Co‐occurrent contigs that conveyed resistance to the beta‐lactam antibiotic class, which contains the most ARGs categorized as clinically relevant, occurred solely on vegetables. Interestingly, across all sample categories, phenicol and quinolone ARGs were only found to co‐occur with MGEs in soils and vegetables and were not detected with MGEs in amendments.

### Comparing abundances of mobile ARGs

Reads matching data were used to quantify relative abundances of the ARGs that were co‐occurrent with MGEs (Fig. 5D). It was observed that several ARGs, especially *tetW*, *vanRO lnuA*, *cpxA* and *mtrA*, displayed strikingly similar relative abundance trends in both the assembled ARG‐MGE contig pool and in the unassembled data. The number of ARG‐MGE contigs correlated with the relative abundance of ARGs, suggesting assembly in replicate is a robust method of assessing co‐occurrent genes (Spearman, rho = 0.17, *p =* 0.01).

## Discussion

The comprehensive, integrated nature of this study provided new insight into the potential for manure‐derived sources of ARGs to perpetuate throughout a vegetable‐production system. Notably, the greenhouse‐scale afforded the opportunity to minimize the influence of external variables and narrow in on the key factors of interest shaping vegetable resistomes. Relative to previous studies (Chen *et al*., 2019; Guron *et al*., 2019), the additional 84 metagenomes included in this analysis also provided balance and replication to the study design, while also allowing the effects of beef‐ and dairy‐derived soil amendments to be more directly compared. An overarching finding was that the relative abundance of total ARGs increased from amendments to soils to vegetables. The detailed analyses and comparisons of various ARG classes and types shared among the different sample categories, along with analysis of clinically relevant ARGs, mobile ARGs and pathogen markers, provided insight into how ARGs move through agroecosystems and which critical management points are best in a position to stem their spread.

### Beef‐derived amendments and antibiotic use differentially impacted resistomes of vegetables grown in loamy sand

The use of beef‐derived amendments tended to raise more concerns than dairy‐derived amendments in terms of the ARG‐based metrics examined in this study. Furthermore, effects of applying amendments derived from beef cattle undergoing antibiotic treatment were apparent relative to antibiotic‐free controls in the loamy sand conditions. Specifically, total ARG relative abundance was elevated and the profile of ARGs was distinct for lettuce and radishes grown in loamy sand soils amended with beef manure with antibiotics compared to the vegetables grown in no antibiotic organic‐amended loamy sand soils. Such effects were not apparent for vegetables grown in the dairy amendment conditions. There were unique ARGs found in antibiotic manure and compost, but these were not detected in downstream soils or vegetables. No unique ARGs were found in vegetable or soil samples as a function of antibiotic administration to the cattle. This suggests that there is some degree of attenuation of ARGs selected in manure specifically as a result of antibiotic use in moving downstream on the farm‐to‐fork continuum. Lack of more striking differences in the antibiotic versus control amendments, particularly for dairy conditions, could be due to generations of antibiotic use and the inherited nature of gut microbiota (Schulfer *et al*., 2018).

### Vegetables carry highest relative abundance of ARGs

Surprisingly, the highest relative abundances of total ARGs were found on radish surfaces, while both radishes and lettuce carried the greatest number of clinically relevant and mobile ARG varieties than soils or amendments. The fact that relative ARG abundances were higher on radishes than on corresponding soils, composts and even manures is consistent with previous reports indicating that the plant rhizosphere can play a role in enhancing dissemination of ARGs from soil to vegetable (Chen *et al*., 2018b; Wang *et al*., 2020a). For example, Pu *et al*. (2019) reported high abundances of clinically relevant ARGs on bok choy grown in soil amended with manure‐derived compost, but not in compost amended soils. Such findings suggest that clinically relevant ARGs actually amplify in soil and further amplify on the vegetable surfaces. This is consistent with the present study.

Given that radishes have greater direct contact with soil, it was expected that resistomes on radishes would be correspondingly more strongly influenced by the soil. However, the opposite trend was found and lettuce resistomes were found to carry more ARGs overlapping with soil (*n* = 72 ARGs) than radish resistomes (*n* = 42 ARGs). Lettuce resistome profiles were also more similar to soils than radish resistomes according to ANOSIM analysis. We were not able to find other studies in the literature that previously examined the differential effects of manure application on the resistome of differing vegetable types, but it is hypothesized that the differences observed here could have to do with the tap root nature of the radish rhizosphere compared to the fine root structure of the lettuce, which affords higher surface area for bacterial colonization.

The fact that lettuce surfaces carried a similar relative abundance of total ARGs, regardless of soil amendment condition, appears to be in conflict with past studies employing qPCR in which it was reported that lettuce fertilized with manure‐derived amendments carried higher relative abundances of ARGs (Wang *et al*., 2015; Zhang *et al*., 2019). This could relate to the wider diversity of ARGs detected by metagenomics. Interestingly, lettuce carried greater relative abundance of taxonomic markers annotated to genera containing produce‐relevant pathogens than radishes, and was even similar in magnitude to manure in this measure. Although the abundance of pathogen‐containing genera markers on lettuce trended towards being higher in manure‐derived versus chemical fertilizer soil amendment conditions, the difference was not statistically significant. Thus, if these annotations represent true pathogens, it is difficult to discern their origin. It is acknowledged that there is substantial uncertainty in confirming pathogen detection by metagenomics, given that virulence typically varies at the strain level.

### Beta‐lactam resistance was the most common clinically relevant antibiotic resistance class on vegetables

Out of the 48 human clinically relevant ARGs detected on vegetable samples, 46 encoded resistance to beta‐lactams. Detection of *blaKPC‐15* and *blaKPC‐10* on radishes was particularly concerning, as these have been identified as rapidly emerging ARGs that convey resistance to ceftazidime, a World Health Organization designated essential medicine (CDC, 2019; World Health Organization, 2019). In addition to shared ARGs (i.e. those found in all samples) being higher in relative abundance on radishes grown with manure or compost amendments, differential ARGs (i.e. those only found uniquely in each of the five sample types) were markedly elevated on both radish and lettuce surfaces. However, because of inherent detection limits in metagenomic sequencing, it cannot be confirmed if many of these clinically relevant ARGs were present at very low abundance in the amendment and soil samples, or if these ARGs originated from airborne sources. Regardless, the results of the current study indicate that the vegetable surface is a unique environment that imposes measurable selection pressure in favour of clinically relevant ARGs.

### Comparison of greenhouse‐ and field‐scale findings

While PCR has been used in prior studies to assess individual ARGs and MGEs across amendment (Fahrenfeld *et al*., 2014), soil (Han *et al*., 2018) and vegetable (Marti *et al*., 2013; Rahube *et al*., 2014; Tien *et al*., 2017; Zhang *et al*., 2019; Zhao *et al*., 2019) microbiomes, no prior study has provided an integrated metagenomic assessment of both different soil and cattle types to track the effect of pre‐harvest conditions on the resistomes at the scale of the present study. A prior field‐scale study (Wind *et al*., 2021) incorporated an experimental design that was analogous to the present study. However, at field‐scale, it was only possible to examine one soil texture (sandy loam), vegetable type (lettuce) and cattle type (dairy). The greenhouse scale of the present study afforded examination of a wider array of factors, while comparing the portion of the experiment that was analogous to the field study afforded some degree of validation. Overall, the similarity of trends relative to those observed in the Wind *et al*. (2021) study was striking. Notably, both studies reported relative abundance of total ARGs increasing from amendment to soil to both lettuce and radish when manure‐derived amendments were applied. Additionally, in the present study, it was possible to observe differential effects of vegetable type, with lettuce carrying lower relative abundances of total ARGs than radishes. In addition to examining both radish and lettuce and a wider range of manure (beef and dairy cattle) and soil textures than was possible than in the Wind *et al*. (2021) study, soil metagenomes were also successfully assembled at much higher rates. This study made it possible to validate trends across scales, supporting the overall conclusion that even with manure management and soil attenuation, there is concern that ARGs can not only persist from farm‐to‐fork but can also be selectively transferred to and enriched on vegetable surfaces. From this study, it was further found that this process varies both by soil and vegetable type.

### Assembly‐based metagenomic analysis of environmental samples

Not only were clinically relevant ARGs highest in relative abundance on vegetable surfaces, but the same was true for mobile ARGs represented by co‐occurrences of ARGs‐MGEs. Interestingly, ARGs that were most frequently found to be associated with MGEs in the soil did not happen to fall into the human clinically relevant category incorporated in this study, as might have been expected based on the understanding that soil is an important reservoir of clinically important ARGs (Cytryn, 2013; Peterson and Kaur, 2018).

The accuracy of short read assembly is known to diminish as a function of sequencing depth and the complexity of the microbial community (Luo *et al*., 2012; Bengtsson‐Palme, 2018). Varying degrees of coverage, which is typical of environmental metagenomes, can also affect comparability of assembled data. In this study, it was expected that the consistency of the experimental conditions (e.g. same manures, composts, soils, etc.) and the assembly approach applied supported relative comparison of the trends. To further support comparability of the data, only co‐occurrences found in multiple replicates were subject to analysis. Notably, good correspondence was observed in comparing quantitative trends in ARGs occurring together with MGEs on assembled contigs and corresponding direct quantification of these ARGs in the unassembled data. Thus, the approach applied here is expected to be robust in terms of identifying ARGs capable of mobilizing across the farm‐to‐fork continuum. However, given the exclusion of singleton ARG‐MGE combinations from the analysis, the approach would not be advised if the goal is to identify emergent ARG‐MGE co‐occurrences. It is also important to consider that vegetable metagenomes are likely better represented in public databases, because their microbiomes have been the subject of more intensive study (given their human health relevance) than soil, manure or compost resistomes (Li *et al*., 2015a). This could have also biased the relative annotations recovered from the three sample categories in this study.

## Conclusions

This integrated metagenomic study across lab‐, microcosm‐ and greenhouse‐scale enabled comprehensive assessment of resistomes from farm to fork and helped to discern and rank critical management points in terms of their relative potential to stem dissemination of antibiotic resistance. Untreated manures posed the highest number of unique ARGs after antibiotic administration, but application to soil generally served to reduce these ARGs below detection. Composting manure reduced pathogen markers, as would be expected, and some measures of ARGs relative to raw manure, but it was concerning that there were more shared clinically relevant ARGs between the compost and vegetables than manure and vegetables, and these were higher on vegetables grown with compost amendments, demonstrating that composting does not alleviate concerns related to antibiotic resistance. Soil and vegetable types were also found to differentially impact the resistomes, suggesting that future guidelines could be tailored depending on the soil and vegetable type. In particular, beef amendments containing antibiotics applied to sandy loam resulted in elevated relative abundance of total ARGs on both vegetables. This study further demonstrated that the vegetable surface itself captures and enriches clinically relevant and mobile ARGs. While radish carried higher total relative abundance of ARGs than lettuce, interestingly, lettuce resistomes contained more ARGs in common with the corresponding soil. In sum, in addition to optimizing the pre‐harvest management practices examined herein, it would also be wise to also consider coupling them with post‐harvest interventions to comprehensively minimize the risk of disseminating antibiotic resistance to consumers.

## Author Contributions

I.K. analysed all data and prepared the manuscript and figures. I.K. and L.W. led the data analysis plan. P.R., G.G., C.C., K.K., M.P. and A.P. designed and led the original experiments and consulted in the integrated analysis presented here. P.R., M.P. and A.P. revised the manuscript.

## Ethics Approval and Consent to Participate

All animal studies were approved by IACUC protocol DASC 13‐145 (PI, K. Knowlton).

## Supporting information


**Appendix S1**. Supporting Information.

## Data Availability

All metagenomic data are available in the NCBI Sequence Read Archive under the SRAs listed in Tables S1 and S2.
