# Supplementary material for: Metagenomic tracking of antibiotic resistance genes through a pre‐harvest vegetable production system: an integrated lab‐, microcosm‐ and greenhouse‐scale analysis
Source: Environ Microbiol. 2022 May 18;24(8):3705–21. doi: 10.1111/1462-2920.16022 (PMC9541739; doi:10.1111/1462-2920.16022)
Supplement: Supplementary file 1 — Appendix S1. Supporting Information. [file EMI-24-3705-s001.docx]

**Supplemental Information:**

Metagenomic Tracking of Antibiotic Resistance Genes through a Pre-harvest Vegetable Production System: An Integrated Lab-, Microcosm-, and Greenhouse-Scale Study

**Authors:** Ishi Keenum^a^, Lauren Wind^b^, Partha Ray ^c^, Giselle Guron^d^, Chaoqi Chen^e^, Katharine Knowlton^f^, Monica Ponder^d^, Amy Pruden ^a*^

^a^ Department of Civil and Environmental Engineering, Virginia Tech, USA

^b^ Department of Biological Systems Engineering, Virginia Tech, USA

^c^ Department of Animal Sciences, School of Agriculture, Policy and Development, Univ. of Reading, Reading RG6 6AR, UK

^d^ Department of Food Science and Technology, Virginia Tech, Blacksburg, VA, USA

^e^ Department of Crop and Soil Environmental Sciences, Virginia Tech, Blacksburg, VA, USA

^f^ Department of Dairy Science, Virginia Tech, Blacksburg, VA, USA

**Table S1: Study Design and Replication. Newly sequenced conditions are in red.**

| **Study Environment** | **Vegetable** | **Time (days)** | **Soil** | **Treatment** | **Cattle** | **Antibiotic dosing in cattle?** | **Number of replicates** |
| --- | --- | --- | --- | --- | --- | --- | --- |
| **Amendment**  **Keenum et al. 2021** | **NA** | **0,4,42** | **NA** | **Small Scale Composting** | **Beef** | **Yes** | **3,2,3** |
|  |  |  |  |  |  | **No** | **3,2,3** |
|  |  |  |  |  | **Dairy** | **Yes** | **3,2,3** |
|  |  |  |  |  |  | **No** | **3,2,3** |
|  |  | **0** |  | **Manure** | **Beef** | **Yes** | **1** |
|  |  |  |  |  |  | **No** | **2** |
|  |  |  |  |  | **Dairy** | **Yes** | **1** |
|  |  |  |  |  |  | **No** | **2** |
|  |  |  |  | **None** | **Chemical Fertilizer** | **No** | **3** |
| **Soil**  **Chen et al. 2019; this study** |  | **1,120** | **Silty Clay Loam** | **Small Scale Composting** | **Beef** | **Yes** | **3,3** |
|  |  |  |  |  |  | **No** | **3,3** |
|  |  |  |  |  | **Dairy** | **Yes** | **3,3** |
|  |  |  |  |  |  | **No** | **3,3** |
|  |  |  |  | **Manure** | **Beef** | **Yes** | **3,3** |
|  |  |  |  |  |  | **No** | **3,3** |
|  |  |  |  |  | **Dairy** | **Yes** | **3,3** |
|  |  |  |  |  |  | **No** | **3,3** |
|  |  |  |  | **None** | **Chemical Fertilizer** | **No** | **3,3** |
|  |  |  | **Loamy Sand** | **Small Scale Composting** | **Beef** | **Yes** | **3,3** |
|  |  |  |  |  |  | **No** | **3,3** |
|  |  |  |  |  | **Dairy** | **Yes** | **3,3** |
|  |  |  |  |  |  | **No** | **3,3** |
|  |  |  |  | **Manure** | **Beef** | **Yes** | **3,3** |
|  |  |  |  |  |  | **No** | **3,3** |
|  |  |  |  |  | **Dairy** | **Yes** | **3,3** |
|  |  |  |  |  |  | **No** | **3,3** |
|  |  |  |  | **None** | **Chemical Fertilizer** | **No** | **3,4** |
| **Vegetable**  **Guron et al. 2019; this study** | **Lettuce** | **34; Harvest** | **Silty Clay Loam** | **Small Scale Composting** | **Beef** | **Yes** | **1** |
|  |  |  |  |  |  | **No** | **1** |
|  |  |  |  |  | **Dairy** | **Yes** | **3** |
|  |  |  |  |  |  | **No** | **3** |
|  |  |  |  | **Manure** | **Beef** | **Yes** | **1** |
|  |  |  |  |  |  | **No** | **1** |
|  |  |  |  |  | **Dairy** | **Yes** | **3** |
|  |  |  |  |  |  | **No** | **3** |
|  |  |  |  | **None** | **Chemical Fertilizer** | **No** | **3** |
|  |  |  | **Loamy Sand** | **Small Scale Composting** | **Beef** | **Yes** | **1** |
|  |  |  |  |  |  | **No** | **1** |
|  |  |  |  |  | **Dairy** | **Yes** | **2** |
|  |  |  |  |  |  | **No** | **2** |
|  |  |  |  | **Manure** | **Beef** | **Yes** | **1** |
|  |  |  |  |  |  | **No** | **1** |
|  |  |  |  |  | **Dairy** | **Yes** | **2** |
|  |  |  |  |  |  | **No** | **2** |
|  |  |  |  | **None** | **Chemical Fertilizer** | **No** | **2** |
|  | **Radish** | **60;Harvest** | **Silty Clay Loam** | **Small Scale Composting** | **Beef** | **Yes** | **3** |
|  |  |  |  |  |  | **No** | **3** |
|  |  |  |  |  | **Dairy** | **Yes** | **4** |
|  |  |  |  |  |  | **No** | **4** |
|  |  |  |  | **Manure** | **Beef** | **Yes** | **3** |
|  |  |  |  |  |  | **No** | **3** |
|  |  |  |  |  | **Dairy** | **Yes** | **4** |
|  |  |  |  |  |  | **No** | **4** |
|  |  |  |  | **None** | **Chemical Fertilizer** | **No** | **3** |
|  |  |  | **Loamy Sand** | **Small Scale Composting** | **Beef** | **Yes** | **3** |
|  |  |  |  |  |  | **No** | **3** |
|  |  |  |  |  | **Dairy** | **Yes** | **4** |
|  |  |  |  |  |  | **No** | **3** |
|  |  |  |  | **Manure** | **Beef** | **Yes** | **3** |
|  |  |  |  |  |  | **No** | **3** |
|  |  |  |  |  | **Dairy** | **Yes** | **4** |
|  |  |  |  |  |  | **No** | **4** |
|  |  |  |  | **None** | **Chemical Fertilizer** | **No** | **3** |

**Table S2: SRAs Accessed from Prior Published Research for Integrated Analysis**

| (Guron et al., 2019) | [SRR7414902](https://trace.ncbi.nlm.nih.gov/Traces/sra?run=SRR7414902) | [SRR7414912](https://trace.ncbi.nlm.nih.gov/Traces/sra?run=SRR7414912) | [SRR7414922](https://trace.ncbi.nlm.nih.gov/Traces/sra?run=SRR7414922) | [SRR7414932](https://trace.ncbi.nlm.nih.gov/Traces/sra?run=SRR7414932) | [SRR7414942](https://trace.ncbi.nlm.nih.gov/Traces/sra?run=SRR7414942) |
| --- | --- | --- | --- | --- | --- |
|  | [SRR7414903](https://trace.ncbi.nlm.nih.gov/Traces/sra?run=SRR7414903) | [SRR7414913](https://trace.ncbi.nlm.nih.gov/Traces/sra?run=SRR7414913) | [SRR7414923](https://trace.ncbi.nlm.nih.gov/Traces/sra?run=SRR7414923) | [SRR7414933](https://trace.ncbi.nlm.nih.gov/Traces/sra?run=SRR7414933) | [SRR7414943](https://trace.ncbi.nlm.nih.gov/Traces/sra?run=SRR7414943) |
|  | [SRR7414904](https://trace.ncbi.nlm.nih.gov/Traces/sra?run=SRR7414904) | [SRR7414914](https://trace.ncbi.nlm.nih.gov/Traces/sra?run=SRR7414914) | [SRR7414924](https://trace.ncbi.nlm.nih.gov/Traces/sra?run=SRR7414924) | [SRR7414934](https://trace.ncbi.nlm.nih.gov/Traces/sra?run=SRR7414934) | [SRR7414944](https://trace.ncbi.nlm.nih.gov/Traces/sra?run=SRR7414944) |
|  | [SRR7414905](https://trace.ncbi.nlm.nih.gov/Traces/sra?run=SRR7414905) | [SRR7414915](https://trace.ncbi.nlm.nih.gov/Traces/sra?run=SRR7414915) | [SRR7414925](https://trace.ncbi.nlm.nih.gov/Traces/sra?run=SRR7414925) | [SRR7414935](https://trace.ncbi.nlm.nih.gov/Traces/sra?run=SRR7414935) | [SRR7414945](https://trace.ncbi.nlm.nih.gov/Traces/sra?run=SRR7414945) |
|  | [SRR7414906](https://trace.ncbi.nlm.nih.gov/Traces/sra?run=SRR7414906) | [SRR7414916](https://trace.ncbi.nlm.nih.gov/Traces/sra?run=SRR7414916) | [SRR7414926](https://trace.ncbi.nlm.nih.gov/Traces/sra?run=SRR7414926) | [SRR7414936](https://trace.ncbi.nlm.nih.gov/Traces/sra?run=SRR7414936) | [SRR7414946](https://trace.ncbi.nlm.nih.gov/Traces/sra?run=SRR7414946) |
|  | [SRR7414907](https://trace.ncbi.nlm.nih.gov/Traces/sra?run=SRR7414907) | [SRR7414917](https://trace.ncbi.nlm.nih.gov/Traces/sra?run=SRR7414917) | [SRR7414927](https://trace.ncbi.nlm.nih.gov/Traces/sra?run=SRR7414927) | [SRR7414937](https://trace.ncbi.nlm.nih.gov/Traces/sra?run=SRR7414937) | [SRR7414947](https://trace.ncbi.nlm.nih.gov/Traces/sra?run=SRR7414947) |
|  | [SRR7414908](https://trace.ncbi.nlm.nih.gov/Traces/sra?run=SRR7414908) | [SRR7414918](https://trace.ncbi.nlm.nih.gov/Traces/sra?run=SRR7414918) | [SRR7414928](https://trace.ncbi.nlm.nih.gov/Traces/sra?run=SRR7414928) | [SRR7414938](https://trace.ncbi.nlm.nih.gov/Traces/sra?run=SRR7414938) | [SRR7414948](https://trace.ncbi.nlm.nih.gov/Traces/sra?run=SRR7414948) |
|  | [SRR7414909](https://trace.ncbi.nlm.nih.gov/Traces/sra?run=SRR7414909) | [SRR7414919](https://trace.ncbi.nlm.nih.gov/Traces/sra?run=SRR7414919) | [SRR7414929](https://trace.ncbi.nlm.nih.gov/Traces/sra?run=SRR7414929) | [SRR7414939](https://trace.ncbi.nlm.nih.gov/Traces/sra?run=SRR7414939) | [SRR7414949](https://trace.ncbi.nlm.nih.gov/Traces/sra?run=SRR7414949) |
|  | [SRR7414910](https://trace.ncbi.nlm.nih.gov/Traces/sra?run=SRR7414910) | [SRR7414920](https://trace.ncbi.nlm.nih.gov/Traces/sra?run=SRR7414920) | [SRR7414930](https://trace.ncbi.nlm.nih.gov/Traces/sra?run=SRR7414930) | [SRR7414940](https://trace.ncbi.nlm.nih.gov/Traces/sra?run=SRR7414940) | [SRR7414950](https://trace.ncbi.nlm.nih.gov/Traces/sra?run=SRR7414950) |
|  | [SRR7414911](https://trace.ncbi.nlm.nih.gov/Traces/sra?run=SRR7414911) | [SRR7414921](https://trace.ncbi.nlm.nih.gov/Traces/sra?run=SRR7414921) | [SRR7414931](https://trace.ncbi.nlm.nih.gov/Traces/sra?run=SRR7414931) | [SRR7414941](https://trace.ncbi.nlm.nih.gov/Traces/sra?run=SRR7414941) | [SRR7414951](https://trace.ncbi.nlm.nih.gov/Traces/sra?run=SRR7414951) |
| (Chen et al., 2019) | SRR8400089 | SRR8710211 | SRR8400092 | SRR8400111 | SRR8710222 |
|  | SRR8400090 | SRR8710212 | SRR8400095 | SRR8400112 | SRR8710225 |
|  | SRR8400091 | SRR8710216 | SRR8400097 | SRR8400113 | SRR8710227 |
|  | SRR8400093 | SRR8710217 | SRR8400098 | SRR8400114 | SRR8710228 |
|  | SRR8400094 | SRR8710218 | SRR8400099 | SRR8400115 | SRR8710229 |
|  | SRR8400096 | SRR8710223 | SRR8400100 | SRR8400116 | SRR8710230 |
|  | SRR8400101 | SRR8710224 | SRR8400102 | SRR8710213 | SRR8710231 |
|  | SRR8400107 | SRR8710226 | SRR8400103 | SRR8710214 | SRR8710232 |
|  | SRR8400109 | SRR8710235 | SRR8400104 | SRR8710215 | SRR8710233 |
|  | SRR8400110 | SRR8710236 | SRR8400105 | SRR8710219 | SRR8710234 |
|  | SRR8400117 | SRR8710237 | SRR8400106 | SRR8710220 | SRR8710238 |
|  | SRR8400118 | SRR8710240 | SRR8400108 | SRR8710221 | SRR8710239 |
| (Keenum et al., 2021) | SRR10980629 | SRR10980625 | SRR10980308 | SRR10980331 | SRR10980310 |
|  | SRR10980630 | SRR10980623 | SRR10980302 | SRR10980636 | SRR10980305 |
|  | SRR10980632 | SRR10980329 | SRR10980637 | SRR10980327 | SRR10980639 |
|  | SRR10980631 | SRR10980330 | SRR10980316 | SRR10980319 | SRR10980318 |
|  | SRR10980622 | SRR10980295 | SRR10980297 | SRR10980323 | SRR10980299 |
|  | SRR10980621 | SRR10980321 | SRR10980645 | SRR10980313 | SRR10980648 |
|  | SRR10980620 | SRR10980311 | SRR10980641 | SRR10980309 | SRR10980643 |
|  | SRR10980634 | SRR10980306 | SRR10980638 | SRR10980304 | SRR10980640 |
|  | SRR10980633 | SRR10980301 | SRR10980317 | SRR10980332 | SRR10980320 |
|  | SRR10980628 | SRR10980307 | SRR10980298 | SRR10980328 | SRR10980300 |
|  | SRR10980627 | SRR10980322 | SRR10980647 | SRR10980324 | SRR10980296 |
|  | SRR10980626 | SRR10980312 | SRR10980642 | SRR10980315 | SRR10980644 |

**Table S3: SRA files for samples sequenced specifically for this study**

| SRR13213324 | SRR13213304 | SRR13213317 | SRR13213268 | SRR13213242 | SRR13213284 | |
| --- | --- | --- | --- | --- | --- | --- |
| SRR13213323 | SRR13213303 | SRR13213316 | SRR13213267 | SRR13213241 | SRR13213283 | |
| SRR13213312 | SRR13213302 | SRR13213315 | SRR13213254 | SRR13213298 | SRR13213281 | |
| SRR13213301 | SRR13213300 | SRR13213314 | SRR13213253 | SRR13213296 | SRR13213280 | |
| SRR13213258 | SRR13213299 | SRR13213313 | SRR13213252 | SRR13213295 | SRR13213279 | |
| SRR13213247 | SRR13213297 | SRR13213311 | SRR13213251 | SRR13213294 | SRR13213278 | |
| SRR13213293 | SRR13213265 | SRR13213310 | SRR13213250 | SRR13213292 | SRR13213277 | |
| SRR13213282 | SRR13213264 | SRR13213309 | SRR13213249 | SRR13213291 | SRR13213276 | |
| SRR13213271 | SRR13213263 | SRR13213308 | SRR13213248 | SRR13213290 | SRR13213275 | |
| SRR13213266 | SRR13213262 | SRR13213307 | SRR13213246 | SRR13213289 | SRR13213274 | |
| SRR13213322 | SRR13213261 | SRR13213306 | SRR13213245 | SRR13213288 | SRR13213273 | |
| SRR13213321 | SRR13213260 | SRR13213305 | SRR13213244 | SRR13213287 | SRR13213272 | |
| SRR13213320 | SRR13213318 | SRR13213269 | SRR13213243 | SRR13213286 | SRR13213270 | |
| SRR13213319 | SRR13213255 | SRR13213285 |  |  |  |  |

**Table S4: ARGs Removed from CARD for analysis**

| CARD Gene Name | Antibiotic Resistance Ontology | Reason for Removing- Sequence variants do not differ by sufficient base pairs to differentiate from sequencing error rate |
| --- | --- | --- |
| Acinetobacter baumannii AbaF | ARO:3004573 | Sequence variant within intrinsic major facility superfamily efflux pump present in all *Acinetobacter baumannii* |
| Acinetobacter baumannii AbaQ | ARO:3004574 | Sequence variant within intrinsic major facility superfamily efflux pump present in all *Acinetobacter baumannii* |
| Acinetobacter baumannii AmpC beta-lactamase | ARO:3003796 | Sequence variant within intrinsic *Acinetobacter baumannii* beta-lactamase |
| Acinetobacter baumannii AmvA | ARO:3004577 | Sequence variant within intrinsic major facility superfamily efflux pump present in all *Acinetobacter baumannii* |
| Agrobacterium fabrum chloramphenicol acetyltransferase | ARO:3004451 | Sequence variant present within *Pseudomonas fluorescens* |
| Brucella suis mprF | ARO:3003772 | Sequence variant present within *Brucella suis* |
| Burkholderia pseudomallei Omp38 | ARO:3004123 | Sequence variant present within *Burkholderia pseudomallei* |
| Campylobacter coli chloramphenicol acetyltransferase | ARO:3004454 | Sequence variant present within *Campylobacter coli*, *Campylobacter jejuni*, *Helicobacter pylori*^,^ *Klebsiella pneumoniae* |
| catII from Escherichia coli K-12 | ARO:3004656 | Sequence variant present within over 20 organisms |
| Chlamydia trachomatis intrinsic murA conferring resistance to fosfomycin | ARO:3003785 | Sequence variant present within *Chlamydia trachomatis* |
| Clostridium perfringens mprF | ARO:3003773 | Sequence variant present within *Clostridia perfringens* |
| Enterobacter cloacae acrA | ARO:3004042 | Sequence variant present within *Enterobacter asburiae*, *Enterobacter cloacae*, *Enterobacter hormaechei*, *Enterobacter kobei*, *Klebsiella pneumoniae* |
| Escherichia coli acrA | ARO:3004043 | Sequence variant present within *Acinetobacter baumannii*, *Citrobacter amalonaticus*, *Citrobacter freundii*, *Citrobacter koseri*, *Citrobacter youngae*, *Enterobacter cloacae*, *Escherichia coli*, *Klebsiella oxytoca*, *Klebsiella pneumoniae*, *Salmonella enterica*, *Serratia marcescens*, *Shigella dysenteriae*, *Shigella flexneri*, *Shigella sonnei*, *Staphylococcus aureus* |
| Escherichia coli ampC1 beta-lactamase | ARO:3004611 | Sequence variant present within *Citrobacter koseri*, *Enterobacter cloacae*, *Enterobacter hormaechei*, *Enterobacter kobei*, *Escherichia coli*, *Klebsiella oxytoca*, *Klebsiella pneumoniae*, *Salmonella enterica*, *Shigella dysenteriae*, *Shigella flexneri*, *Shigella sonnei*, *Staphylococcus aureus*, *Stenotrophomonas maltophilia* |
| Escherichia coli emrE | ARO:3004039 | Sequence variant present within *Acinetobacter baumannii*, *Escherichia coli*, *Klebsiella oxytoca*, *Klebsiella pneumoniae*, *Shigella dysenteriae*, *Shigella flexneri*, *Shigella sonnei*, *Staphylococcus aureus* |
| Escherichia coli mdfA | ARO:3001328 | Sequence variant present within *Acinetobacter baumannii*, *Citrobacter amalonaticus*, *Citrobacter freundii*, *Citrobacter koseri*, *Citrobacter youngae*, *Enterobacter cloacae*, *Enterobacter hormaechei*, *Escherichia coli*, *Klebsiella oxytoca*, *Klebsiella pneumoniae*, *Listeria monocytogenes*, *Proteus mirabilis*, *Pseudomonas aeruginosa*, *Salmonella enterica*, *Shigella dysenteriae*, *Shigella flexneri*, *Shigella sonnei*, *Staphylococcus aureus*, *Stenotrophomonas maltophilia* |
| Klebsiella pneumoniae acrA | ARO:3004041 | Sequence variant present within *Acinetobacter baumannii*, *Enterobacter cloacae*, *Enterobacter hormaechei*, *Escherichia coli*, *Klebsiella aerogenes*, *Klebsiella pneumoniae*, *Raoultella planticola*, *Salmonella enterica*, *Stenotrophomonas maltophilia* |
| Klebsiella pneumoniae KpnE | ARO:3004580 | Sequence variant present within *Citrobacter amalonaticus*, *Citrobacter freundii*, *Citrobacter koseri*, *Citrobacter youngae*, *Enterobacter asburiae*, *Enterobacter cloacae*, *Enterobacter hormaechei*, *Enterobacter kobei*, *Escherichia coli*, *Klebsiella aerogenes*, *Klebsiella oxytoca*, *Klebsiella pneumoniae*, *Raoultella planticola*, *Salmonella enterica*, *Shigella dysenteriae*, *Shigella flexneri*, *Shigella sonnei*, *Stenotrophomonas maltophilia* |
| Klebsiella pneumoniae KpnF | ARO:3004583 | Sequence variant present within *Citrobacter amalonaticus*, *Citrobacter freundii*, *Citrobacter koseri*, *Citrobacter youngae*, *Enterobacter asburiae*, *Enterobacter cloacae*, *Enterobacter hormaechei*, *Enterobacter kobei*, *Escherichia coli*, *Klebsiella aerogenes*, *Klebsiella oxytoca*, *Klebsiella pneumoniae*, *Proteus mirabilis*, *Proteus penneri*, *Proteus vulgaris*, *Providencia rettgeri*, *Providencia stuartii*, *Raoultella planticola*, *Salmonella enterica*, *Serratia liquefaciens*, *Serratia marcescens*, *Serratia odorifera*, *Shigella dysenteriae*, *Shigella flexneri*, *Shigella sonnei*, *Stenotrophomonas maltophilia*, *Yersinia enterocolitica*, *Yersinia pestis* |
| Klebsiella pneumoniae KpnG | ARO:3004588 | Sequence variant present within *Acinetobacter baumannii*, *Enterobacter asburiae*, *Enterobacter cloacae*, *Escherichia coli*, *Klebsiella aerogenes*, *Klebsiella oxytoca*, *Klebsiella pneumoniae*, *Proteus mirabilis*, *Raoultella planticola*, *Serratia marcescens*, *Stenotrophomonas maltophilia* |
| Klebsiella pneumoniae KpnH | ARO:3004597 | Sequence variant present within *Citrobacter amalonaticus*, *Citrobacter freundii*, *Citrobacter koseri*, *Enterobacter asburiae*, *Enterobacter cloacae*, *Enterobacter hormaechei*, *Enterobacter kobei*, *Escherichia coli*, *Haemophilus influenzae*, *Haemophilus parainfluenzae*, *Klebsiella aerogenes*, *Klebsiella oxytoca*, *Klebsiella pneumoniae*, *Morganella morganii*, *Proteus mirabilis*, *Proteus penneri*, *Proteus vulgaris*, *Providencia rettgeri*, *Providencia stuartii*, *Raoultella planticola*, *Salmonella enterica*, *Serratia liquefaciens*, *Serratia marcescens*, *Serratia odorifera*, *Shigella dysenteriae*, *Shigella flexneri*, *Shigella sonnei*, *Yersinia enterocolitica*, *Yersinia pestis* |
| Klebsiella pneumoniae OmpK37 | ARO:3004122 | Sequence variant present within *Acinetobacter baumannii*, *Enterobacter cloacae*, *Enterococcus faecium*, *Escherichia coli*, *Klebsiella aerogenes*, *Klebsiella pneumoniae*, *Salmonella enterica*, *Serratia marcescens*, *Shigella flexneri* |
| Lactobacillus reuteri cat-TC | ARO:3002671 | Sequence variant present within *Campylobacter*, *Enterococcus faecium*, *Escherichia coli*, *Jeotgalibaca*, *Klebsiella pneumoniae*, *Salmonella enterica*, *Serratia marcescens*, *Staphylococcus aureus*, *Streptococcus*, *Streptococcus pneumoniae* |
| Listeria monocytogenes mprF | ARO:3003770 | Sequence variant present within *Listeria monocytogenes* |
| Pseudomonas aeruginosa catB7 | ARO:3002679 | Sequence variant present within *Acinetobacter baumannii*, *Pseudomonas aeruginosa*, *Pseudomonas fluorescens* |
| Pseudomonas aeruginosa CpxR | ARO:3004054 | Sequence variant present within *Pseudomonas aeruginosa*, *Pseudomonas fluorescens* |
| Pseudomonas aeruginosa emrE | ARO:3004038 | Sequence variant present within *Acinetobacter baumannii*, *Neisseria meningitidis*, *Pseudomonas aeruginosa*, *Pseudomonas fluorescens*, *Stenotrophomonas maltophilia*, *Streptococcus pneumoniae* |
| Pseudomonas aeruginosa soxR | ARO:3004107 | Sequence variant present within *Klebsiella pneumoniae*, *Pseudomonas aeruginosa*, *Pseudomonas fluorescens*, *Pseudomonas putida*, *Stenotrophomonas maltophilia* |
| Staphylococcus aureus FosB | ARO:3004661 | Sequence variant present within *Staphylococcus aureus* |
| Staphylococcus aureus norA | ARO:3004667 | Sequence variant present within *Enterococcus faecalis*, *Klebsiella pneumoniae*, *Staphylococcus aureus* |
| Streptococcus agalactiae mprF | ARO:3003774 | Sequence variant present within *Listeria monocytogenes*, *Streptococcus agalactiae* |
| Streptococcus suis chloramphenicol acetyltransferase | ARO:3004455 | Sequence variants present in *Campylobacter coli*, *Campylobacter jejuni*, *Enterococcus faecium*, *Klebsiella pneumoniae*, *Salmonella enterica*, *Serratia marcescens*, *Staphylococcus aureus*, *Streptococcus agalactiae*, *Streptococcus pneumoniae*, *Streptococcus pyogenes* |
| vga(E) Staphylococcus cohnii | ARO:3004715 | Sequence variant from *Staphylococcus aureus* |
| Vibrio cholerae varG | ARO:3004289 | Sequence variant from *Vibrio cholerae* intrinsic in the *var* operon |

**Table S5: Sub-set of Clinically-Relevant ARGs (Keenum et al., 2021; Majeed et al., 2021)**

| CARB-1 | CARB-3 | CTX-M-104 | CTX-M-116 | CTX-M-132 | CTX-M-152 | CTX-M-22 | CARB-17 |
| --- | --- | --- | --- | --- | --- | --- | --- |
| CARB-10 | CARB-4 | CTX-M-105 | CTX-M-117 | CTX-M-134 | CTX-M-155 | CTX-M-23 | CARB-18 |
| CARB-12 | CARB-5 | CTX-M-106 | CTX-M-12 | CTX-M-136 | CTX-M-156 | CTX-M-24 | CARB-19 |
| CARB-14 | CARB-6 | CTX-M-107 | CTX-M-121 | CTX-M-137 | CTX-M-157 | CTX-M-25 | CARB-2 |
| CARB-16 | CARB-7 | CTX-M-108 | CTX-M-122 | CTX-M-139 | CTX-M-158 | CTX-M-26 | CARB-20 |
| CTX-M-34 | TEM-150 | CTX-M-109 | CTX-M-123 | CTX-M-14 | CTX-M-159 | CTX-M-27 | CARB-21 |
| CTX-M-35 | TEM-63 | CTX-M-11 | CTX-M-124 | CTX-M-141 | CTX-M-16 | CTX-M-28 | CARB-22 |
| CTX-M-36 | CTX-M-1 | CTX-M-110 | CTX-M-125 | CTX-M-142 | CTX-M-160 | CTX-M-29 | CARB-23 |
| CTX-M-37 | CTX-M-10 | CTX-M-111 | CTX-M-126 | CTX-M-144 | CTX-M-17 | CTX-M-3 | CARB-8 |
| CTX-M-38 | CTX-M-100 | CTX-M-112 | CTX-M-129 | CTX-M-147 | CTX-M-19 | CTX-M-30 | CARB-9 |
| CTX-M-39 | CTX-M-101 | CTX-M-113 | CTX-M-13 | CTX-M-148 | CTX-M-2 | CTX-M-31 | VIM-2 |
| CTX-M-4 | CTX-M-102 | CTX-M-114 | CTX-M-130 | CTX-M-15 | CTX-M-20 | CTX-M-32 | VIM-20 |
| CTX-M-40 | CTX-M-103 | CTX-M-115 | CTX-M-131 | CTX-M-151 | CTX-M-21 | CTX-M-33 | TEM-137 |
| CTX-M-41 | CTX-M-76 | GES-3 | OXA-100 | OXA-15 | OXA-203 | OXA-249 | TEM-138 |
| CTX-M-42 | CTX-M-77 | GES-4 | OXA-101 | OXA-150 | OXA-204 | OXA-25 | TEM-139 |
| CTX-M-43 | CTX-M-78 | GES-5 | OXA-104 | OXA-16 | OXA-205 | OXA-250 | TEM-141 |
| CTX-M-44 | CTX-M-79 | GES-6 | OXA-106 | OXA-160 | OXA-206 | OXA-251 | TEM-188 |
| CTX-M-45 | CTX-M-8 | GES-7 | OXA-107 | OXA-161 | OXA-207 | OXA-253 | TEM-189 |
| CTX-M-46 | CTX-M-80 | GES-8 | OXA-108 | OXA-162 | OXA-208 | OXA-254 | TEM-19 |
| CTX-M-47 | CTX-M-81 | GES-9 | OXA-109 | OXA-163 | OXA-209 | OXA-255 | VIM-11 |
| CTX-M-48 | CTX-M-82 | KPC-10 | OXA-11 | OXA-164 | OXA-21 | OXA-256 | TEM-142 |
| CTX-M-49 | CTX-M-83 | KPC-11 | OXA-110 | OXA-165 | OXA-210 | OXA-257 | TEM-143 |
| CTX-M-5 | CTX-M-84 | KPC-12 | OXA-111 | OXA-166 | OXA-211 | OXA-258 | TEM-144 |
| CTX-M-50 | CTX-M-85 | KPC-13 | OXA-112 | OXA-167 | OXA-212 | OXA-26 | TEM-145 |
| CTX-M-51 | CTX-M-86 | KPC-14 | OXA-113 | OXA-168 | OXA-213 | OXA-27 | TEM-146 |
| CTX-M-52 | CTX-M-87 | KPC-15 | OXA-114a | OXA-169 | OXA-214 | OXA-278 | TEM-147 |
| CTX-M-53 | CTX-M-88 | KPC-16 | OXA-115 | OXA-17 | OXA-215 | OXA-28 | TEM-148 |
| CTX-M-54 | CTX-M-89 | KPC-17 | OXA-116 | OXA-170 | OXA-216 | OXA-29 | TEM-149 |
| CTX-M-55 | CTX-M-9 | KPC-19 | OXA-117 | OXA-171 | OXA-217 | OXA-3 | TEM-92 |
| CTX-M-56 | CTX-M-90 | KPC-2 | OXA-118 | OXA-172 | OXA-219 | OXA-309 | TEM-192 |
| CTX-M-58 | CTX-M-91 | KPC-22 | OXA-119 | OXA-173 | OXA-22 | OXA-31 | TEM-193 |
| CTX-M-59 | CTX-M-92 | KPC-3 | OXA-12 | OXA-174 | OXA-223 | OXA-312 | TEM-194 |
| CTX-M-6 | CTX-M-93 | KPC-4 | OXA-120 | OXA-175 | OXA-224 | OXA-313 | TEM-195 |
| CTX-M-60 | CTX-M-94 | KPC-5 | OXA-121 | OXA-176 | OXA-225 | OXA-314 | TEM-196 |
| CTX-M-61 | CTX-M-95 | KPC-6 | OXA-128 | OXA-177 | OXA-226 | OXA-315 | TEM-197 |
| CTX-M-62 | CTX-M-96 | KPC-7 | OXA-129 | OXA-178 | OXA-228 | OXA-316 | TEM-198 |
| CTX-M-63 | CTX-M-98 | KPC-8 | OXA-13 | OXA-179 | OXA-229 | OXA-317 | TEM-199 |
| CTX-M-64 | CTX-M-99 | KPC-9 | OXA-130 | OXA-18 | OXA-23 | OXA-32 | TEM-2 |
| CTX-M-65 | GES-1 | MCR-1 | OXA-131 | OXA-180 | OXA-230 | OXA-320 | TEM-90 |
| CTX-M-66 | GES-10 | mecA | OXA-132 | OXA-181 | OXA-231 | OXA-322 | TEM-49 |
| CTX-M-67 | GES-11 | NDM-1 | OXA-133 | OXA-182 | OXA-232 | OXA-323 | TEM-52 |
| CTX-M-68 | GES-12 | NDM-10 | OXA-134 | OXA-183 | OXA-233 | OXA-324 | TEM-53 |
| CTX-M-69 | GES-13 | NDM-12 | OXA-136 | OXA-184 | OXA-235 | OXA-325 | TEM-54 |
| CTX-M-7 | GES-14 | NDM-13 | OXA-137 | OXA-19 | OXA-236 | OXA-326 | TEM-55 |
| CTX-M-71 | GES-15 | NDM-14 | OXA-138 | OXA-192 | OXA-237 | OXA-327 | TEM-57 |
| CTX-M-72 | GES-16 | NDM-17 | OXA-139 | OXA-194 | OXA-239 | OXA-328 | TEM-59 |
| CTX-M-74 | GES-17 | NDM-2 | OXA-14 | OXA-195 | OXA-24 | OXA-329 | TEM-6 |
| CTX-M-75 | GES-18 | NDM-3 | OXA-141 | OXA-196 | OXA-240 | OXA-33 | TEM-60 |
| OXA-347 | GES-19 | NDM-4 | OXA-142 | OXA-197 | OXA-241 | OXA-330 | TEM-30 |
| OXA-348 | GES-2 | NDM-5 | OXA-143 | OXA-198 | OXA-242 | OXA-331 | TEM-33 |
| OXA-349 | GES-20 | NDM-6 | OXA-144 | OXA-199 | OXA-243 | OXA-332 | TEM-34 |
| OXA-35 | GES-21 | NDM-7 | OXA-145 | OXA-2 | OXA-244 | OXA-333 | TEM-4 |
| OXA-350 | GES-22 | NDM-8 | OXA-146 | OXA-20 | OXA-245 | OXA-334 | TEM-40 |
| OXA-351 | GES-23 | NDM-9 | OXA-147 | OXA-200 | OXA-246 | OXA-335 | TEM-42 |
| OXA-352 | GES-24 | OXA-1 | OXA-148 | OXA-201 | OXA-247 | OXA-338 | TEM-43 |
| OXA-353 | GES-26 | OXA-10 | OXA-149 | OXA-202 | OXA-248 | OXA-34 | TEM-45 |
| OXA-354 | OXA-397 | OXA-71 | QnrS8 | SHV-149 | SHV-26 | SHV-69 | TEM-47 |
| OXA-355 | OXA-398 | OXA-72 | QnrS9 | SHV-15 | SHV-27 | SHV-7 | TEM-48 |
| OXA-356 | OXA-4 | OXA-73 | SHV-1 | SHV-150 | SHV-28 | SHV-70 | TEM-178 |
| OXA-357 | OXA-415 | OXA-74 | SHV-100 | SHV-151 | SHV-29 | SHV-71 | TEM-182 |
| OXA-358 | OXA-418 | OXA-75 | SHV-101 | SHV-152 | SHV-2A | SHV-72 | TEM-183 |
| OXA-359 | OXA-42 | OXA-76 | SHV-102 | SHV-153 | SHV-3 | SHV-73 | TEM-184 |
| OXA-36 | OXA-420 | OXA-77 | SHV-103 | SHV-154 | SHV-30 | SHV-74 | TEM-185 |
| OXA-360 | OXA-421 | OXA-78 | SHV-104 | SHV-155 | SHV-31 | SHV-75 | TEM-186 |
| OXA-361 | OXA-422 | OXA-79 | SHV-105 | SHV-156 | SHV-32 | SHV-76 | TEM-187 |
| OXA-362 | OXA-423 | OXA-80 | SHV-106 | SHV-157 | SHV-33 | SHV-77 | TEM-190 |
| OXA-363 | OXA-424 | OXA-82 | SHV-107 | SHV-158 | SHV-34 | SHV-78 | TEM-191 |
| OXA-365 | OXA-425 | OXA-83 | SHV-108 | SHV-159 | SHV-35 | SHV-79 | TEM-67 |
| OXA-366 | OXA-426 | OXA-84 | SHV-109 | SHV-16 | SHV-36 | SHV-8 | TEM-132 |
| OXA-368 | OXA-43 | OXA-85 | SHV-11 | SHV-160 | SHV-37 | SHV-80 | TEM-133 |
| OXA-37 | OXA-435 | OXA-86 | SHV-110 | SHV-161 | SHV-38 | SHV-81 | TEM-134 |
| OXA-370 | OXA-45 | OXA-87 | SHV-111 | SHV-162 | SHV-39 | SHV-82 | TEM-135 |
| OXA-371 | OXA-454 | OXA-88 | SHV-112 | SHV-163 | SHV-40 | SHV-83 | TEM-136 |
| OXA-374 | OXA-46 | OXA-89 | SHV-119 | SHV-164 | SHV-41 | SHV-84 | TEM-15 |
| OXA-375 | OXA-47 | OXA-9 | SHV-12 | SHV-165 | SHV-42 | SHV-85 | TEM-168 |
| OXA-376 | OXA-48 | OXA-90 | SHV-120 | SHV-167 | SHV-43 | SHV-86 | TEM-169 |
| OXA-377 | OXA-49 | OXA-91 | SHV-121 | SHV-168 | SHV-44 | SHV-89 | TEM-17 |
| OXA-378 | OXA-5 | OXA-92 | SHV-122 | SHV-172 | SHV-45 | SHV-9 | TEM-171 |
| OXA-379 | OXA-50 | OXA-93 | SHV-123 | SHV-173 | SHV-46 | SHV-92 | TEM-176 |
| OXA-380 | OXA-51 | OXA-94 | SHV-124 | SHV-178 | SHV-48 | SHV-93 | TEM-177 |
| OXA-381 | OXA-53 | OXA-95 | SHV-125 | SHV-179 | SHV-49 | SHV-94 | TEM-220 |
| OXA-382 | OXA-54 | OXA-96 | SHV-126 | SHV-18 | SHV-5 | SHV-95 | TEM-24 |
| OXA-383 | OXA-55 | OXA-97 | SHV-127 | SHV-180 | SHV-50 | SHV-96 | TEM-26 |
| OXA-384 | OXA-56 | OXA-98 | SHV-128 | SHV-181 | SHV-51 | SHV-97 | TEM-28 |
| OXA-385 | OXA-57 | OXA-99 | SHV-129 | SHV-182 | SHV-52 | SHV-98 | TEM-29 |
| OXA-386 | OXA-58 | QnrA1 | SHV-13 | SHV-183 | SHV-53 | SHV-99 | TEM-3 |
| OXA-387 | OXA-59 | QnrA2 | SHV-133 | SHV-185 | SHV-55 | TEM-1 | TEM-84 |
| OXA-388 | OXA-60 | QnrA3 | SHV-134 | SHV-186 | SHV-56 | TEM-10 | TEM-85 |
| OXA-389 | OXA-61 | QnrA4 | SHV-135 | SHV-187 | SHV-57 | TEM-101 | TEM-86 |
| OXA-390 | OXA-62 | QnrA5 | SHV-137 | SHV-188 | SHV-59 | TEM-102 | TEM-87 |
| OXA-391 | OXA-63 | QnrA6 | SHV-14 | SHV-189 | SHV-6 | TEM-104 | TEM-88 |
| TEM-112 | OXA-64 | QnrA7 | SHV-140 | SHV-19 | SHV-60 | TEM-105 | TEM-89 |
| TEM-113 | OXA-65 | QnrS1 | SHV-141 | SHV-2 | SHV-61 | TEM-106 | TEM-151 |
| TEM-114 | OXA-66 | QnrS2 | SHV-142 | SHV-20 | SHV-62 | TEM-107 | VIM-25 |
| TEM-115 | OXA-67 | QnrS3 | SHV-143 | SHV-21 | SHV-63 | TEM-108 | TEM-20 |
| TEM-116 | OXA-68 | QnrS4 | SHV-144 | SHV-22 | SHV-64 | TEM-109 | TEM-68 |
| TEM-117 | OXA-69 | QnrS5 | SHV-145 | SHV-23 | SHV-65 | TEM-11 | VIM-23 |
| TEM-118 | OXA-7 | QnrS6 | SHV-147 | SHV-24 | SHV-66 | TEM-110 | TEM-91 |
| TEM-12 | OXA-70 | QnrS7 | SHV-148 | SHV-25 | SHV-67 | TEM-111 | TEM-94 |
| TEM-120 | TEM-155 | TEM-207 | TEM-72 | VIM-27 | VIM-38 | VIM-26 | TEM-95 |
| TEM-121 | TEM-156 | TEM-208 | TEM-73 | VIM-28 | VIM-39 | TEM-7 | TEM-96 |
| TEM-122 | TEM-157 | TEM-209 | TEM-75 | VIM-29 | VIM-4 | TEM-70 | vanA |
| TEM-123 | TEM-158 | TEM-21 | TEM-76 | VIM-3 | VIM-42 | TEM-71 | VIM-1 |
| TEM-124 | TEM-159 | TEM-211 | TEM-77 | VIM-30 | VIM-43 | TEM-201 | VIM-10 |
| TEM-125 | TEM-16 | TEM-213 | TEM-78 | VIM-31 | VIM-5 | TEM-205 | VIM-14 |
| TEM-126 | TEM-160 | TEM-214 | TEM-79 | VIM-32 | VIM-6 | TEM-206 | VIM-15 |
| TEM-127 | TEM-162 | TEM-215 | TEM-8 | VIM-33 | VIM-7 | TEM-152 | VIM-16 |
| TEM-128 | TEM-163 | TEM-216 | TEM-80 | VIM-34 | VIM-8 | TEM-153 | VIM-17 |
| TEM-129 | TEM-164 | TEM-217 | TEM-81 | VIM-35 | VIM-9 | TEM-154 | VIM-18 |
| TEM-130 | TEM-166 | TEM-219 | TEM-82 | VIM-36 | VIM-24 | VIM-12 | VIM-19 |
| TEM-131 | TEM-167 | TEM-22 | TEM-83 | VIM-37 | TEM-93 | VIM-13 |  |

**Table S5: Clinically Relevant ARGs detected in each sample media**

| Sample Media | Clinically relevant ARGs |
| --- | --- |
| Amendment | CARB-10, CARB-14, CARB-3, CARB-4, CARB-5, CARB-8, CTX-M-122, CTX-M-130, CTX-M-131, CTX-M-38, MCR-3.5, MCR-4.2, MCR-5.1, MCR-5.2, MCR-8.1, MCR-9.1, MECR1, OXA-119, OXA-164, OXA-18, OXA-209, OXA-21, OXA-22, OXA-226, OXA-229, OXA-236, OXA-274, OXA-278, OXA-29, OXA-296, OXA-3, OXA-31, OXA-334, OXA-347, OXA-357, OXA-361, OXA-363, OXA-37, OXA-443, OXA-46, OXA-486, OXA-50, OXA-60, OXA-724, SHV-22, SHV-70, TEM-102, TEM-126, VANA, VIM-37 |
| Soil | CARB-10, CARB-14, CARB-3, CARB-4, CARB-5, CTX-M-122, CTX-M-151, CTX-M-159, CTX-M-19, CTX-M-56, CTX-M-68, CTX-M-74, KPC-10, MCR-3.3, MCR-3.5, MCR-4.2, MCR-5.1, MCR-5.2, MCR-7.1, MCR-8.1, MCR-9.1, MECA, OXA-119, OXA-134, OXA-15, OXA-164, OXA-18, OXA-20, OXA-205, OXA-209, OXA-21, OXA-212, OXA-226, OXA-229, OXA-235, OXA-236, OXA-258, OXA-27, OXA-275, OXA-278, OXA-282, OXA-29, OXA-296, OXA-3, OXA-300, OXA-31, OXA-320, OXA-347, OXA-356, OXA-357, OXA-358, OXA-360, OXA-361, OXA-362, OXA-363, OXA-37, OXA-420, OXA-43, OXA-46, OXA-47, OXA-486, OXA-50, OXA-53, OXA-60, OXA-664, OXA-96, SHV-70, TEM-102, TEM-116, TEM-126, TEM-196, vanA |
| Vegetable | CARB-3, CARB-12, CARB-5, CTX-M-121, CTX-M-123, CTX-M-13, CTX-M-130, CTX-M-131, CTX-M-134, CTX-M-144, CTX-M-151, CTX-M-155, CTX-M-4, CTX-M-56, CTX-M-68, CTX-M-74, CTX-M-76, CTX-M-8, CTX-M-88, CTX-M-95, KPC-10, KPC-15, MCR-1.1, MCR-3.5, MCR-4.2, MCR-5.1, MCR-5.2, MCR-6.1, MCR-7.1, MCR-8.1, MCR-9.1, MECA, MECC, MECR1, OXA-119, OXA-12, OXA-134, OXA-143, OXA-15, OXA-18, OXA-205, OXA-209, OXA-21, OXA-211, OXA-212, OXA-22, OXA-226, OXA-228, OXA-229, OXA-230, OXA-236, OXA-243, OXA-257, OXA-258, OXA-270, OXA-274, OXA-275, OXA-278, OXA-287, OXA-288, OXA-29, OXA-296, OXA-3, OXA-330, OXA-333, OXA-347, OXA-348, OXA-355, OXA-362, OXA-364, OXA-37, OXA-373, OXA-417, OXA-427, OXA-43, OXA-443, OXA-45, OXA-464, OXA-486, OXA-50, OXA-53, OXA-59, OXA-60, OXA-62, OXA-724, OXA-90, SHV-41, SHV-52, SHV-81, TEM-102, TEM-112, TEM-116, TEM-126, TEM-176, TEM-186, TEM-34, TEM-59, TEM-72, vanA |

**Table S6: Contig Co-occurrence Counts**

| Sample Media | Soil Type | Antibiotic Addition | Sample_id | Unique Contig Co-occurences |
| --- | --- | --- | --- | --- |
| Amendment | None | No | Manure_0 | 27 |
|  |  |  | Compost_0 | 11 |
|  |  |  | Compost_4 | 19 |
|  |  |  | Compost_42 | 105 |
|  |  | Yes | Manure_0 | 16 |
|  |  |  | Compost_0 | 18 |
|  |  |  | Compost_4 | 52 |
|  |  |  | Compost_42 | 71 |
| Soil | Loamy Sand | No | Manure_1 | 31 |
|  |  |  | Manure_120 | 30 |
|  |  |  | Compost_1 | 91 |
|  |  |  | Compost_120 | 47 |
|  |  | Yes | Manure_1 | 75 |
|  |  |  | Manure_120 | 14 |
|  |  |  | Compost_1 | 111 |
|  |  |  | Compost_120 | 40 |
|  | Silty Clay Loam | No | Manure_1 | 5 |
|  |  |  | Manure_120 | 8 |
|  |  |  | Compost_1 | 28 |
|  |  |  | Compost_120 | 24 |
|  |  | Yes | Manure_1 | 15 |
|  |  |  | Compost_1 | 5 |
|  |  |  | Compost_120 | 3 |
| Vegetable | Loamy Sand | No | Lettuce_Manure | 16 |
|  |  |  | Lettuce_Compost | 77 |
|  |  |  | Radish_Chemical | 2 |
|  |  |  | Radish_Manure | 267 |
|  |  |  | Radish_Compost | 146 |
|  |  | Yes | Lettuce_Manure | 23 |
|  |  |  | Lettuce_Compost | 61 |
|  |  |  | Radish_Manure | 768 |
|  |  |  | Radish_Compost | 133 |
|  | Silty Clay Loam | No | Lettuce_Manure | 46 |
|  |  |  | Radish_Chemical | 5 |
|  |  |  | Radish_Manure | 316 |
|  |  |  | Radish_Compost | 221 |
|  |  | Yes | Radish_Manure | 191 |
|  |  |  | Radish_Compost | 200 |

**Figure S1:** Average relative abundance (normalized to the 16S rRNA gene) of total ARGs annotated to CARD v3.0.7 (minimum identity = 80%, 25 amino acid length, e-value cutoff 1 x 10^-10^) with point mutation genes removed (Table S3). The relative abundance of ARGs on lettuce grown in loamy sand (n = 12) was significantly greater than in lettuce grown in silty clay loam (n =8) (p = 0.02, Wilcoxon) Error bars indicate the standard deviation. Number of replicates for each condition are indicated in Figure 1. Yes = antibiotic condition, No = no antibiotic control.

**
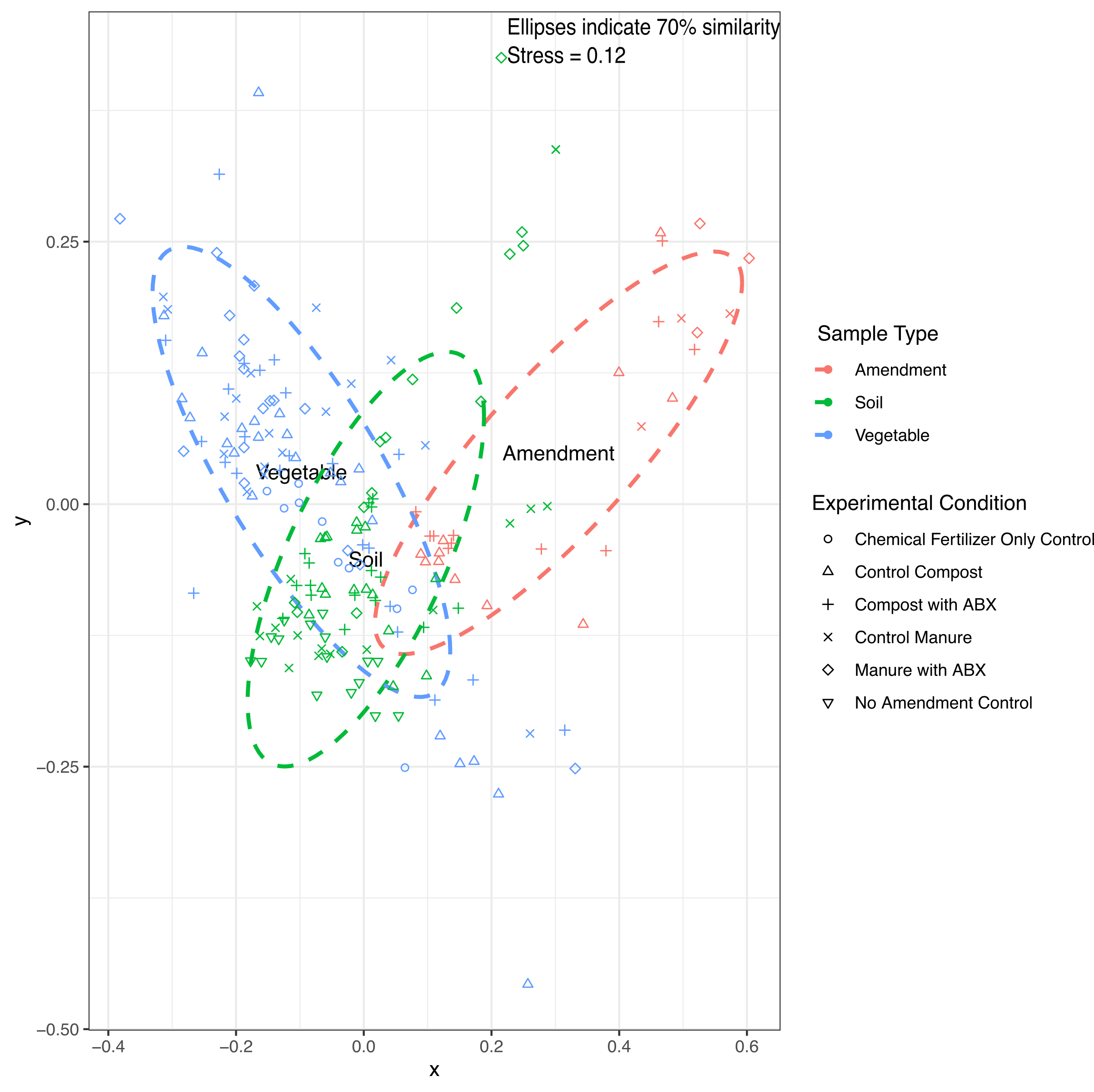
**

**Figure S2:** NMDS of ARGs contained within Vegetable, Amendment, Soil metagenomes determined using the Bray Curtis dissimilarity matrix. Ellipses indicate 70% similarity.

**Figure S3:** ARGs detected in all samples by drug class. Average relative abundance (normalized to the 16S rRNA gene) of total ARGs annotated to CARD v3.0.7 (minimum identity = 80%, 25 amino acid length, e-value cutoff 1 x 10^-10^) with point mutation genes removed (Table S3). Yes = antibiotic condition, No = no antibiotic control. See Figure 3 for categorization of ARGs and lumped analysis of these conditions.

**Figure S4:** Analysis of ARGs that were uniquely identified in each experimental condition. Average relative abundance (normalized to the 16S rRNA gene) of total ARGs annotated to CARD v3.0.7 (minimum identity = 80%, 25 amino acid length, e-value cutoff 1 x 10^-10^) with point mutation genes removed (Table S3). Yes = antibiotic condition, No = no antibiotic control. See Figure 3 for categorization of ARGs and lumped analysis of these conditions.

References:

Chen, C., Pankow, C.A., Oh, M., Heath, L.S., Zhang, L., Du, P. et al. (2019) Effect of antibiotic use and composting on antibiotic resistance gene abundance and resistome risks of soils receiving manure-derived amendments. *Environment International* **128**: 233-243.

Guron, G.K., Arango-Argoty, G., Zhang, L., Pruden, A., and Ponder, M.A. (2019) Effects of Dairy Manure-Based Amendments and Soil Texture on Lettuce-and Radish-Associated Microbiota and Resistomes. *mSphere* **4**: e00239-00219.

Keenum, I., Williams, R.K., Ray, P., Garner, E.D., Knowlton, K.F., and Pruden, A. (2021) Combined effects of composting and antibiotic administration on cattle manure–borne antibiotic resistance genes. *Microbiome* **9**: 81.

Majeed, H.J., Riquelme, M.V., Davis, B.C., Gupta, S., Angeles, L., Aga, D.S. et al. (2021) Evaluation of Metagenomic-Enabled Antibiotic Resistance Surveillance at a Conventional Wastewater Treatment Plant. *Frontiers in Microbiology* **12**: 1048.
